# The Open Studio Approach to Art Therapy: A Systematic Scoping Review

**DOI:** 10.3389/fpsyg.2020.568042

**Published:** 2020-10-20

**Authors:** Daniela Finkel, Michal Bat Or

**Affiliations:** The School of Creative Arts Therapies, Faculty of Social Welfare and Health Sciences, University of Haifa, Haifa, Israel

**Keywords:** open studio, art therapy, scoping review, art-based, community-based

## Abstract

This research encompasses a systematic scoping review of literature and research pertaining to the open studio approach to art therapy, which originated with the work of artists in psychiatric hospitals in the 1940's. As art therapy became a profession, it sought recognition by adopting theories from other therapeutic disciplines. Today, however, there is an increase in the prevalence of studio practice that emphasizes art as the core of the therapeutic work; moreover, contemporary art therapy approaches even venture beyond the traditional definition of the profession to the realm of social action. Consequently, open studio practice has become more widespread and is currently implemented in many different contexts among a wide range of populations. The purpose of this research was to accurately map out world literature and research on the open studio approach to art therapy as well as identify relevant publications and main themes. Therefore, the systematic scoping review was not restricted to specific periods, languages, settings, or populations. Publications were identified through a rigorous, replicable, and extensive search of international literature in data bases and hand searches in art therapy journals; in addition, special efforts were made to locate unpublished research and literature. Data was charted using a tool developed by the researchers, based on the review questions. Results indicate that most of the literature relating to the open studio approach dates from the 1990's: only a few earlier publications were found. Over the last decade, the literature has grown in volume in comparison to previous decades, reflecting an increasing prevalence of the open studio approach. This growing mass of publications reflects an historic development in the field of art therapy. This research also identifies core principles as well as a wide range of variations on the open studio model, in addition to unique characteristics that vary according to context and therapeutic approach. It explores open studio practice within different settings and populations and pinpoints gaps of knowledge that can indicate the need for further research.

## Introduction

The open studio approach to art therapy positions art as the focus of the therapeutic work (Moon, 2016). It originates from the early days of the art therapy profession, when artists, influenced by humanistic approaches to psychiatry, brought the studio into psychiatric hospitals (Hogan, 2001). As the art therapy profession evolved, it adopted theories from clinical psychology in order to gain recognition; Allen (1992) termed this the “clinification syndrome.” Following developments in recent decades in the field of art therapy, open studio practices have become more diverse and more prevalent in a wider range of contexts (Kapitan, 2008). This article presents a systematic scoping review of existing literature and research pertaining to the open studio approach to art therapy. It seeks to map out variations and common core principles of the practice as well as unique aspects that emerge from a range of contexts and therapeutic approaches.

## What is the Open Studio Approach to Art Therapy?

In 1946, Edward Adamson began to work as an “art master” at the Netherne Psychiatric Hospital in the south of London. The hospital's head psychiatrist, Dr. E. Cunningham Dax, asked him to open an art studio to research the role of art in diagnosis and treatment (Hogan, 2001). His vague job description did not fall within the realms of occupational therapist or art teacher, and this perhaps gave him an opportunity to offer the patients a space where they could express themselves through artistic creation and a deferential and non-invasive approach that respected the patient's autonomy (Pitman, 2016). Mary Huntoon also began her work the same year at the Winter VA Hospital in Topeka, Kansas. Both an artist and art teacher, she established a studio at the hospital that served as a space in which patients could express themselves through artmaking. In 1947, she received a grant to research the “studio as laboratory for research observation,” and even organized hospital exhibitions of her patients' artwork. The guiding principle of her work was that artistic creation has therapeutic value (Wix, 2000).

The open studio reflects the art as therapy approach, in which the artistic processes and outcomes are central to the therapeutic work and incorporate healing qualities (Kramer, 2000), as opposed to other approaches, in which art serves as a foundation for an interpretative process based on theories from various disciplines. The main features of the open studio approach emphasize artistic creation and provide the venue and conditions for this process. The artmaking process is not directed or moderated by the facilitators, and the sessions, deliberately longer than clinical sessions, enable profound engagement and sufficient time for the creative process to evolve. The nature of the space and its organization are vitally important to the establishment of a welcoming and enabling atmosphere. The emphasis is on the individual's creative process in a group setting that represents communality (Allen, 1995; Malchiodi, 1995; McNiff, 1995; Shapiro, 2014; Moon, 2016). Allen (1983) conceived the term “open studio” to describe a model of group art therapy that she implemented in a short-term psychiatric unit; the model offered participants the freedom to choose to take part and engage in artmaking, and she herself would participate in the artmaking alongside them. These ideas emerged out of Allen's exploration of her roots as an artist and her vision of the centrality of art in the therapeutic process (Allen, 1995). Moon (2016) explores the meaning of the word “open” as it is used in “open studio,” and suggests that this openness is reflected in different aspects, such as the freedom to choose the materials; the process that is neither steered or moderated; and the open and optional invitation that allows the participant to choose whether to participate and for how long. This openness is also present in community-based models, which are open to anyone who wants to participate, and no screening or intake processes are involved. Openness can also refer to the facilitation process, in which facilitators are full partners as they engage in artmaking alongside participants (Moon, 2016).

Open studio practice allows participants to work at their own pace, regulate their interactions with other participants according to their abilities, and use the group and the space according to their needs. This approach incorporates aspects of a community life where individuals live together and separately (Deco, 1998). The community aspect, together with the space for individual expression and the therapeutic benefits of artistic creation, make this model also relevant beyond the clinical setting. Over time, the open studio has moved from within the confines of the psychiatric hospital and is implemented in diverse forms and in many settings. For example, Kapitan (2008) describes the use of art as a means for healing communities in regions hit by catastrophe and trauma; in prisons; and when dealing with children at risk, She challenges the present definition of art therapy, and suggests expanding it to social settings where the healing powers of art can be put to good use. The studio approach, with its inherent communal aspect, is especially suitable for these contexts. Based on the literature, the open studio approach is used in many different social spaces. These range from an open studio project for youth at risk described by Block et al. (2005) and a community-based model of a studio for people with disabilities (Vick and Sexton-Radek, 2008) to other models of community studios such as “art hives,” which are neighborhood spaces for creative work in Canada (Timm-Bottos and Reilly, 2015). Therapeutic models based on the open studio approach are also operated in educational spaces: Henley (1995) asserts the importance of a studio space for art therapy in a school for special education; Heller (2015), who studied an open studio at a school found that participation in an open studio enhanced children's reflective abilities, empowered them and increased their sense of self-efficacy.

As the open studio model has been used in a wide variety of contexts and settings, it has been adapted to the unique needs of different populations. This diversity, enabled by the flexible nature of this approach (reflected in the name “open studio”) underlines the importance of its closer examination, as well as the identification of common core principles and unique features that have emerged from specific contexts or therapeutic approaches.

## The Open Studio in Research

Many studies have underlined the contribution of artmaking to an improved quality of life. One study, in which adults were invited to create freely in an open studio-like space, found that the artmaking reinforced positive feelings, reduced negativity and significantly enhanced the sense of self-efficacy (Kaimal and Ray, 2017). Artmaking in rehabilitative settings builds confidence, encourages creativity, and contributes to a greater sense of social belonging and well-being, as well as an enhanced quality of life (Sapouna and Pamer, 2014). Art-based interventions were also found to be helpful in processing grief (Finn, 2003), encouraging expression among children who had experienced trauma (Klorer, 2005), and coping with crisis (Tyson and Baffour, 2004). Open studio research, which is growing and expanding in recent years, focuses for the most part on the therapeutic efficacy of the approach. For instance, a mixed method study that examined the influence of an open studio group on the mood of patients who were hospitalized in an acute care psychiatric setting (Chiu et al., 2015) revealed that negative moods decreased following participation in the group. Griffith, Seymour & Goldberg's mixed method study (2015) demonstrates the positive therapeutic effect of a community-based open studio setting on homeless individuals; it examines the effect of participation in an open studio setting and an art cooperative on life achievement, and suggests art therapy as a therapeutic tool for addressing financial and psycho-social needs of homeless individuals. The researchers found a positive significant correlation between participation in the open studio group and life achievement, i.e., finding a job, rehabilitation, finding housing, initiative and more. In addition, participants who took part in the open studio and sold their artwork through the art cooperative showed a greater increase in life achievement than participants who only attended the open studio. The sale of artwork created in the studio emphasizes the open studio's rehabilitative role, in addition to its therapeutic goals.

The studio approach, which allows for expression of healthy aspects of individual personality, is especially relevant today in light of the prevalence of post-modern approaches to mental health, such as the recovery approach (Anthony, 1993) and the well-being theory (Seligman, 2011), which discard the medical approach and emphasize health and well-being. Moreover, as Allen (1992) asserts, art, as the focus of therapeutic work, anchors the work of the art therapist and reduces the clinification process that characterizes the work of art therapists who work in mental health care systems.

## The Systematic Scoping Review

Systematic scoping reviews entail the systematic search and summary of qualitative and quantitative studies, case studies, and articles written on a certain subject (Peters et al., 2015). They are useful when a large body of literature has not yet been reviewed, especially when existing materials are heterogeneous in nature. Systematic scoping reviews identify, evaluate and summarize findings pertaining to all the relevant literature, and, in this way, summarize all current knowledge and make it accessible to professionals in this field (Peters et al., 2015).

The systematic scoping review includes a structured and systematic search for articles and studies according to a pre-determined protocol of search criteria and a systematic representation of the findings (Tricco et al., 2018).

As this is the first systematic review of the open studio approach to art therapy, the systematic scoping review method was chosen because of its methodical and comprehensive compilation of information. While systematic scoping reviews mainly consist of research-based literature, we decided to include theoretical literature as well, as it documents the development and principles of the open studio approach. Through this integrated review of quantitative and qualitative research and information found in both theoretical and empirical literature, we sought to gain a profound understanding of the phenomenon under examination.

## The Present Study

The purpose of this study was to map out and examine the scope of the open studio approach to art therapy as reflected in professional, clinical and research literature. Variations on the model were also reviewed, as well as common characteristics and unique differences. The review also identifies trends and aspects that need further research. For the purpose of the study, a research question was formulated based on PCC (population, concept, content) key concepts (Tricco et al., 2018). These concepts were chosen because this research examined existing variations of the open studio approach as implemented among different populations and settings.

The present review related to the following questions:

1. Based on the literature, what do we know about the open studio approach to art therapy?
2. What kinds of publications/studies exist on the open studio approach to art therapy?
3. In what contexts and venues is this approach implemented and among what populations?
4. What are the common factors and the unique difference in the way the approach is implemented in all its variations?
5. Are there changes in the open studio approach over time, and how are they reflected in the writing on this subject?

## Method

The purpose of the search process was to identify existing publications pertaining to the open studio approach to art therapy. The search was carried out in a systematic, replicable and comprehensive manner from May to September 2019, and the database search was conducted on May 31st. The search encompassed leading data bases: PUBMED; PsycINFO; Scopus; Cochrane; Cinahl; Eric; ProQuest Dissertations; World Cat; ULI; and Web of Science; as well as a manual search of art therapy journals in English such as The Arts in Psychotherapy Journal; Art Therapy Journal; International Art Therapy Journal; The Canadian Art Therapy Journal; Journal of Clinical Art Therapy; and journals in Israel such as Beyn Hamilim and The Academic Journal of Creative Arts Therapies. The search was carried out using three search parameters: title, abstract and key words. The search strategy included the following terms: “open studio” or “studio approach” or “community art studio” or “art hive” or “social action art studio” or “studio art therapy” or “therapeutic studio” or “community-based art studio.” In Hebrew, the keyword was “studio patuach” which is the Hebrew translation for “open studio.” In addition, a search for gray material was conducted (unpublished research or articles, lectures given at conferences, etc.) by reaching out to leading experts in the field and scanning websites of professional associations, conference proceedings, and reference lists of findings that came up in the systematic search. The search was conducted in English and Hebrew. Our decision was based on several considerations. First, our assumption was that most of the existing literature is in English. Secondly, the researchers' knowledge of these languages enabled a more efficient search and screening process. Thirdly, we assumed that most of the articles published in other languages include an abstract in English, and we would be able to translate them if, based on this abstract, they would potentially be included in the review.

The findings were screened by two researchers who applied the pre-determined inclusion criteria and worked independently of one another, using the “Covidence” software for systematic review management. The researchers resolved disagreements on publication selection by discussing the disagreements and reaching a consensus.

### Inclusion and Exclusion Criteria

Based on the research strategy, quantitative, qualitative, and mixed method studies were included, as were action research, case studies, articles and chapters from books about the open studio approach to art therapy. According to the systematic scoping review method, published findings were included as well as unpublished full texts. Findings relating to a wide range of settings and populations were included (different age groups, normative as well as clinical populations, and populations from different cultures). In addition, as this is the first systematic scoping review of this field, the search was not restricted to specific years. Finally, as was explained in detail above, we included publications in any language that came up in the systematic search.

Exclusion criteria for this review were applied to findings that did not reference art therapy or the open studio approach. However, we did include findings relating to the studio approach though they referenced models that did not define themselves as art therapy *per se* because of political or ideological reasons; in essence, however, these models did adhere to the principles of the open studio approach to art therapy. These include, for example, disability studios (Lentz, 2008). Similarly, keeping with systematic scoping review protocol, studies and relevant articles that are currently being written, and no full text is yet available, were not included.

### Data Charting

Extraction of relevant information was done in a systematic manner according to PRISMA-ScR (Preferred Reporting Items for Systematic reviews and Meta-Analyses extension for Scoping Reviews) (Tricco et al., 2018), and using a tool developed by the researchers based on the research questions. The data extraction process included classification of findings according to (a) type of publication (research, case studies, theoretical material); (b) research method (e.g., qualitative, quantitative, mixed method); (c) date of publication; (d) characteristics of population (age, type of pathology/normative); (e) setting (clinical, community, educational, therapist training); (f) intervention type (facilitators engaging in artmaking alongside participants or not; exhibition or no exhibition of art work; facilitators' perception of open studio participants as artist, participant or client; length and frequency of sessions; make up of group (regular participants or continuously changing group); (g) country; (h) results of studies.

## Results

**Figure 1:**
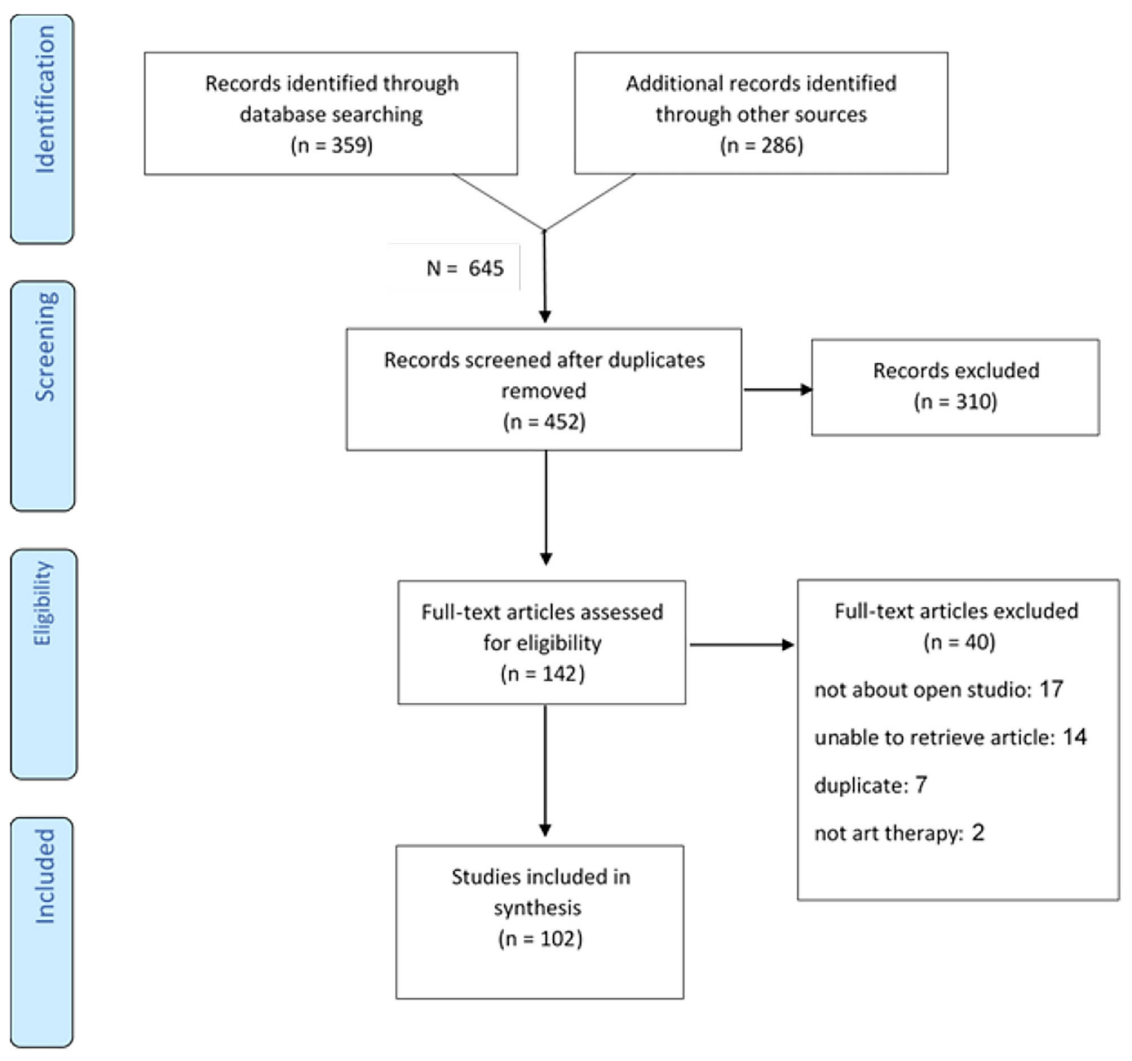
Systematic search according to the PRISMA statement methodology, extension for Scoping Reviews (PRISMA-ScR) (Tricco et al., 2018).

The PRISMA flow diagram (Figure 1) presents the systematic search and screening process. Three hundred fifty-nine publications were found as a result of the systematic search of data bases, and 286 publications were found following a manual search of journals, a scan for gray literature on websites of professional associations in Israel and abroad, and a review of conference proceedings. Altogether, 645 publications were found. Out of these, 193 findings were duplicates, so 452 articles remained to be screened. The first screening, conducted by the two researchers independently of one another, was done according to title and abstract. At this point, 310 articles were discarded from the review following application of the protocol's inclusion criteria, and 142 publications remained. During the second screening stage, the inclusion criteria were applied to the full texts of the publications. Fourty publications were excluded for the following reasons: 17 did not pertain to the studio approach; the text of 14 findings was inaccessible; 7 were duplicates; 2 were not about art therapy. Fourteen publications (mainly theses) included in the first stage of the screening were not included in the review, because the full text was inaccessible. Several attempts were made to obtain these full texts, including requests submitted to the libraries of the relevant universities and colleges that were turned down. In addition, we attempted to obtain contact information for the authors. Finally, 102, i.e., 16% of the publications identified through the first systematic search were found suitable to be included in the review.

### Publication Characteristics

**Figure 2:**
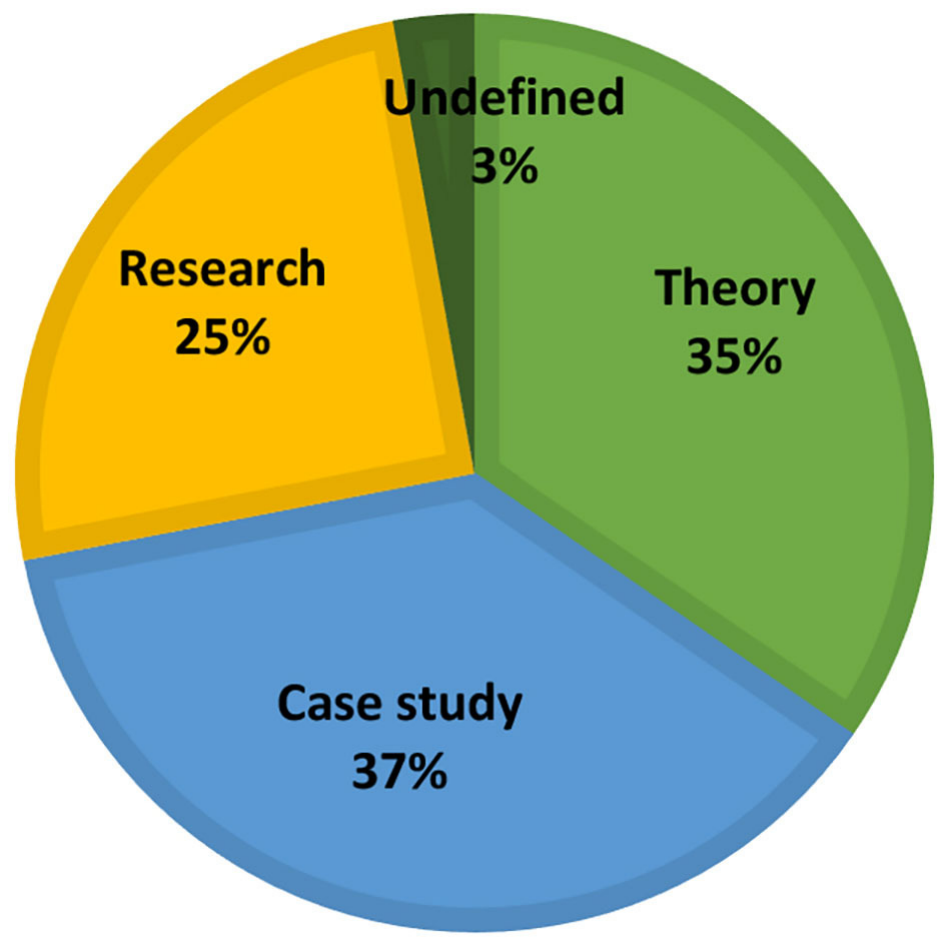
Distribution of results according to publication type.

**Figure 3:**
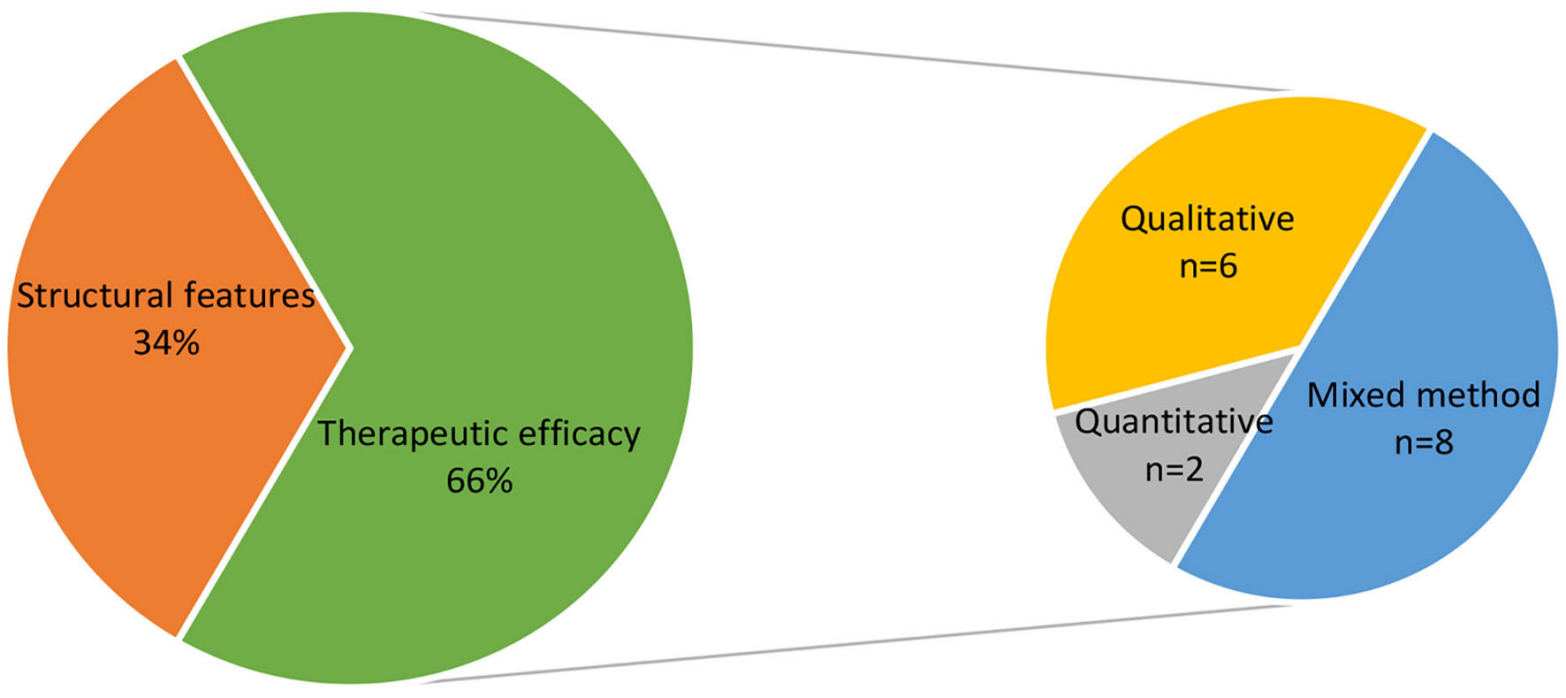
Distribution of results according to research type.

The 645 findings that resulted from the systematic search process include articles and studies from art therapy, art, art education, architecture, television, theater, music, computers and more. Out of these, 102 fit the inclusion criteria that define the studio approach to art therapy. Figure 2 shows the distribution of results according to publication type. Thirty seven% of the publications are case studies, 35% are articles on theoretical aspects of the studio approach, and 25% are studies. 3% of the publications do not belong to one of these categories and include book reviews or proposals for potential open studio programs. 66% of the studies conducted on the open studio approach deal with its therapeutic efficacy, and 34% deal with structural features, studio conditions and how the intervention takes place (Figure 3). Among the studies that examined therapeutic efficacy, most of the studies are mixed-method studies (*n* = 8), qualitative studies and action studies (*n* = 6), and a minority are quantitative studies (*n* = 2) (Figure 3). As shown in Table 1, these quantitative and mixed method studies were predominantly based on self-report questionnaires such as distress scales, mood questionnaires, questionnaires that measure sense of self-efficacy, and more. Three of the mixed method and quantitative studies were quasi-experimental, i.e., measurements were conducted before and after the intervention and there was no control group. The qualitative information in the mixed-method and qualitative studies was generated using semi-structured interviews and analysis based on a narrative approach; observations and transcriptions of sessions; documentation and analysis of artwork using art-based research methods; participants reflections on their creative work; and more.

**Table 1:** Studies that examined therapeutic efficacy.

**Author(s)**	**Type of study**	**N**	**Population (age in years)**	**Open studio setting**	**Data collection and analysis**	**Main results**
Allan et al. (2015)UK	Mixed	13	Adults (22–65) coping with acute mental illness in transition from acute to community mental health care.	The open studio operates in an art organization in the city center. Affiliated to NHS in Britain. Based on the principles of the recovery approach. Weekly, 2-h meetings for a period of 8–13 months, moderated by two facilitators. Meetings include an introduction, artmaking and time for sharing. During the group sessions, facilitators circulate among participants and ask them about their daily lives.	Self-report questionnaires at the beginning and end of the program: The Clinical Outcomes in Routine Evaluation screening measure (CORE-10) Warwick-Edinburgh Mental Well-Being Scale (WEMWBS) Social inclusion measure Qualitative data was gathered by means of a semi-structured interview with each participant at the end of the program.	Quantitative: 9 out of 13 participants demonstrated significantly lower distress levels and/or increase in quality of life at end of program as compared to beginning. Qualitative: Participants reported that the group contributed significantly to their quality of life.
Chiu et al. (2015)CAN	Mixed	36	Adults (23–79) in acute psychiatric states and hospitalized in the psychiatric department.	The Open Studio is situated in the psychiatric department of Toronto General Hospital. It operates once a week for 2 h and is facilitated by an art therapist and a student. The study was conducted over a period of half a year. The group was open to anyone including staff and interns and there was no need for a referral. It offers a creative experience in a community atmosphere; the door is open, and you can join or leave freely.	Data was collected using a self-report questionnaire that relates to mood at a specific point of time. A POMS-B questionnaire was filled out by participants before and after the group sessions. A true-false questionnaire was administered to the clients at the end of the therapeutic session in order to collect data about their open studio experience. In addition, the study includes two vignettes.	Quantitative: Significant reduction of negative moods after open studio session. 81% of participants reported they would be interested in participating in a similar community group, once they are released from hospital. Qualitative: Participants reported that the group allowed them to express experiences that they were unable to express in words and increased their sense of community belonging.
Czamanski-Cohen (2010)ISR	Qualitative	4	Girls (13–14) who were evacuated from their homes in Gush Katif during implementation of the Disengagement Plan.	Open studio in a girls' school, facilitated by one art therapist; 1 weekly session during the school year.	Case study that uses the collaborative inquiry approach to art therapy research to analyze and document conversation, interactions, use of the space and artwork in an open studio. Artwork was analyzed using a compositional and psychoanalytic approach. Semi-structured interview of participants on their experience of the studio and the presentation of their work.	Analysis of observations and interviews reveals that the open studio allows normalization of feelings about abnormal or chaotic situations. Artmaking allowed the processing of memories and reinforced a sense of community belonging. The enjoyable process helped evacuees cope with depression and anhedonia.
Czamanski-Cohen (2012)ISR	Qualitative	5	Adults (ages not specified) suffering from cancer who are undergoing chemotherapy.	The open studio held weekly sessions facilitated by one art therapist in the Support Center for Cancer Patients.	Case study that makes use of narrative analysis based on the collaborative inquiry approach to art therapy research by documenting conversation, use of the space and art in open studio sessions. Semi-structured interviews that include reflective observation of artwork and participants' experience of the open studio. Shared process of reflection involving the interviewee, on analyzed data from the interview.	Observations and interviews reveal that artmaking helped participants who were conflicted regarding their treatments. Artmaking supported the decision-making process and boosted courage. Art helped patients connect to their inner selves and find answers to their questions. Art also helped examine past medical decisions.
Glinzak (2016)USA	Quantitative	73	Adult (above 18) cancer patients in treatment or in follow-up care.	The efficacy of various art therapy interventions was examined, including an open studio operating in the oncology department of a general hospital. The sessions were open, took place twice a month and lasted 5.5 h; participants came and went as they pleased.	Analysis of self-report (distress thermometer) questionnaires that participants filled out before and after art therapy in four different settings: individual intervention in the chemotherapy treatment clinic; individual intervention beside the patient's bed in the oncology department; long-term individual therapy; open studio at hospital.	All interventions were found to be effective in reducing stress. Out of the four settings, the open studio was the most effective in reducing stress.
Griffith et al. (2015)USA	Mixed	78	Homeless adults (ages not specified).	An open studio in a community center that serves the homeless, combined with a gallery that sells artwork. Open every day. The research was conducted over a period of 1 year.	Observations and documentation of changes in six areas according to categories of life achievement (Prescott et al., 2008) combined with vignettes from therapy sessions. Associations between frequency of attendance of group sessions and increase in life achievement were examined; a comparison was made between life achievement of participants who took part in the open studio only and those of participants who took part in both the studio and art cooperative that sells artwork.	Quantitative: a significant positive correlation was found between participation in the group and an increase in life achievement such as finding a job, rehabilitation, finding housing, initiative, etc. In addition, participants who took part both in the open studio as well as the gallery for sale of artwork demonstrated a more meaningful increase in life achievements than the participants who only took part in the open studio. Qualitative: Vignettes based on work with various patients.
Heller (2015)ISR	Mixed	16	Children (11–12) in primary school.	Open studio in a primary school, 20 weekly meetings. Each group has 4 participants. Research conducted on 4 groups.	The qualitative data was collected from observations, reflective content analysis, and a semi-structured interview. The quantitative data was collected by identifying coping styles using a 6-part story and self-report questionnaires relating to the concept of academic self-efficacy (Sherir and Maddux, 1982) and the concept of social self-efficacy (Fan and Mak, 1998; Matsushima and Shiomi, 2003),	Quantitative: Participation in the open studio resulted in an increased sense of self-efficacy and ability to cope with problems. Qualitative: reflective practice mirrors a wider range of cognitive skills.
Howells and Zelnik (2009)USA	Qualitative	20	Adults (24–75). Half suffer from psychiatric disorders and half did not report any psychiatric disorders.	Community-based open studio operating in a psychiatric rehabilitation center. The open studio operated in the city center in a building that was not affiliated with the rehabilitation center. The studio holds art classes and includes a gallery and workspace. Open to all community members. The research was conducted over the course of 1 year.	Action study that uses ethnographic tools, such as in-depth semi structured interviews, observations and an observation journal kept by the researchers.	Interviews and content of observation journals revealed that art making allowed the participants to assume new identities and roles. A community of artists was created, and art was perceived as a bridge to the community-at-large. Participants reported that the outcome, and not only the process, was important.
Kaimal et al. (2017)CAN	Quantitative	29	Healthy adults (19–67).	An open art therapy studio in a university. Each participant had two individual sessions with an art therapist. One meeting was held in an open studio format where materials were laid out and the participant engaged in unguided artmaking. In the other meeting. participants chose a coloring sheet and used either colored pencils or markers to do the coloring.	An experimental study in which each participant had one session of each kind. Positive and Negative Affect Schedule (PANAS); General Self Efficacy Scale (GSE) and Perceived Stress Scale (PSS) self-report questionnaires were administered before and after the sessions.	Both interventions led to a higher positive affect, creative experience and sense of self efficacy. The open studio contributed more than individual coloring in terms of positive affect, creative experience and sense of self efficacy, and was equal to individual coloring in regard to reduction of stress, especially among younger participants.
Kaimal and Ray (2017)USA	Mixed	39	Healthy adults (18–59).	45-min individual session in an open studio format held in an art therapy studio in a university. The session was facilitated by an art therapist.	Quasi-experimental study (measures taken before and after intervention, no control group) PANAS, a validated standardized measure (Watson et al., 1988) and the validated General Self-Efficacy Scale (GSES; Schwarzer and Jerusalem, 1995) self-report questionnaires measuring positive and negative affect and sense of self-efficacy administered before and	Quantitative: significant reduction of negative affect and increase of positive affect and sense of self-efficacy after artmaking in the open studio. Qualitative: The artwork included a variety of themes: nature, people, activities and abstract exploration of colors and shapes.
					after the session. In addition, participants summed up their experiences of artmaking at the end of the session and wrote a narrative summary of their artwork.	
Maselli (1998)USA	Mixed	16	Healthy adults (21–82).	A community-based open studio operating in a church. Weekly, 2-h sessions over the course of 10.5 months. Two groups of adults with 8 participants each, facilitated by an art therapist. Participants could attend as they please, entrance was free.	Quasi-experimental (measures taken before and after intervention, no control group) as well as naturalistic- ethnographic study. Included observations and documentation of written and verbal comments of the participants, analysis of artwork and self-report questionnaires that measure levels of depression and preferred use of time. Beck Depression Inventory; Luscher Quick Color Test Evaluations; Oinebell Time-Values Inventory.	Quantitative: Reduction of depression levels and changes in priorities regarding health and well-being. Qualitative: Participants reported changes in the way they use their free time. In addition, they reported that the open studio experience was meaningful to them and generated self-exploration with a focus on self-development.
Ourso (2016)USA	Qualitative	10	Youth and adults (15–50) no specific characteristics.	Privately operated, community-based open studio situated in city center. Research was conducted over a period of 6 weeks.	Action study that examined the emotional effects of participation in the open studio. Uses self-report questionnaires based on study participants' feedback that examined the emotional effects of participation in the open studio. These questionnaires were filled out by participants before and after the course of the study. In addition, use of semi-structured interviews, analysis of artwork and observations of researcher.	Participants reported a decrease in stress levels, improved moods and an increase in energy levels.
Phoenix-Beck (2018)USA	Mixed	18	Adults above the age of 65.	Open Studio in a community center for seniors. Research conducted over a period of 6 weeks.	Quantitative data generated by Ottawa Mood Scale self-report questionnaire. Qualitative data collected by means of demographic questionnaires and one-word descriptions relating to filled out questionnaire.	Quantitative: increase in mood level. Qualitative: better communication skills that can contribute to quality of life of participating seniors.
Piot and Plante (2009)CAN	Mixed	35	Adults suffering from cancer (no specific reference to age).	Open studio near a hospital for oncology patients. The research was conducted over a period of 7 months.	Self-report questionnaires (name not noted) and semi-structured interview.	Quantitative: Reinforced sense of control and increased self-esteem. Qualitative: Participants reported that the open studio was a refuge and it reinforced their sense of belonging.
Stokrocki et al. (2004)USA	Qualitative	3	Homeless women (no specific reference to age) who had experienced domestic violence.	Open studio for homeless women established in the researcher's home as part of a research project.	Action study including interviews.	Interviews with women reveal that artmaking in the open studio provides important social connections, meaning and strengthens self-esteem.
Thompson (2016)USA	Qualitative	10	Adults psychiatric patients diagnosed with a severe mental illness (no specific reference to age).	12 meetings of an open studio over a 6-week period that included presentation of work in a gallery at the Community Mental Health Center. Participants were patients that were in transition from hospitalization to community mental health care.	Action study combined with art-based research and narrative analysis of semi-structured interviews. The purpose was to explore the researchers/partners experience of the transformative effect of art.	Participant interviews reveal that artmaking in the open studio promotes an artist identity through the formation of a new sense of self and the discovery of empowering new self-narratives. Symptoms were reduced as was the dependence on negative aspects of the psychiatric narratives.

**Figure 4:**
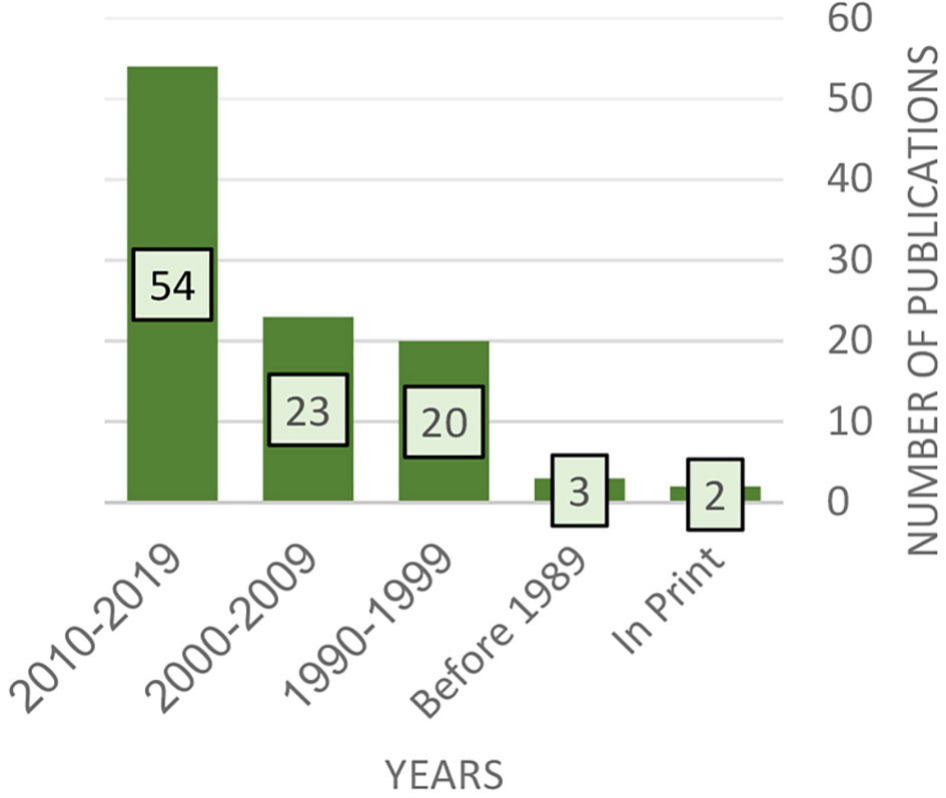
Distribution of results according to publication year.

**Figure 5:**
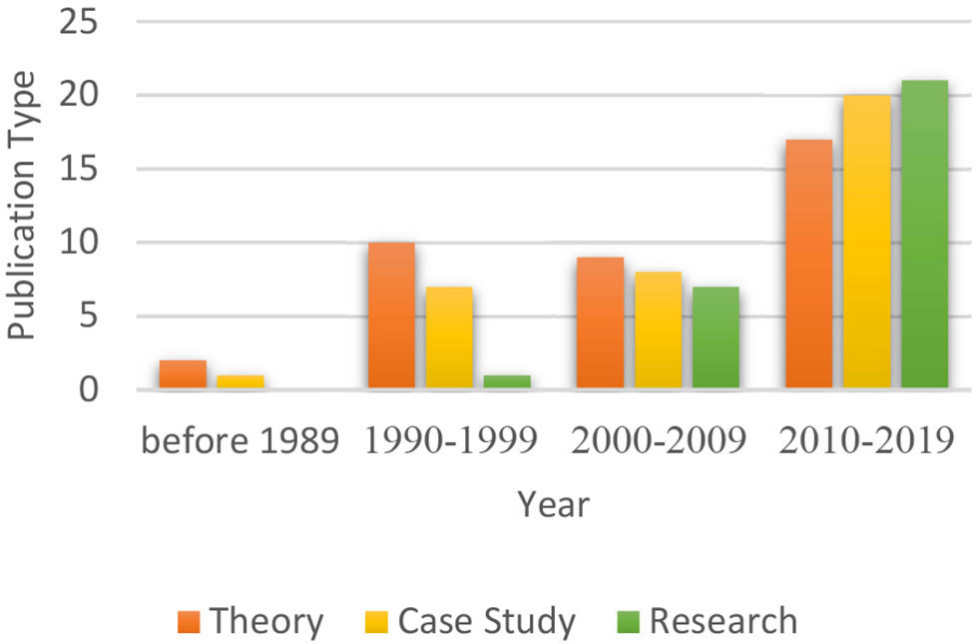
Distribution of publication type according to publication Year.

As Figure 4 reveals, most of the existing publications on the open studio approach are from the 1990's or later, and this volume of work has been growing steadily since. The number of publications, which has almost doubled in the last decade, can indicate the increasing popularity of this method. Initially, the literature was mainly theoretical in nature; in recent years, however, the number of research-based publications has grown steadily and even exceeds that of theory-based texts (see Figure 5). This could indicate that the open studio approach is increasingly accepted as part of art therapy practice and that there is growing academic interest in this model.

**Figure 6:**
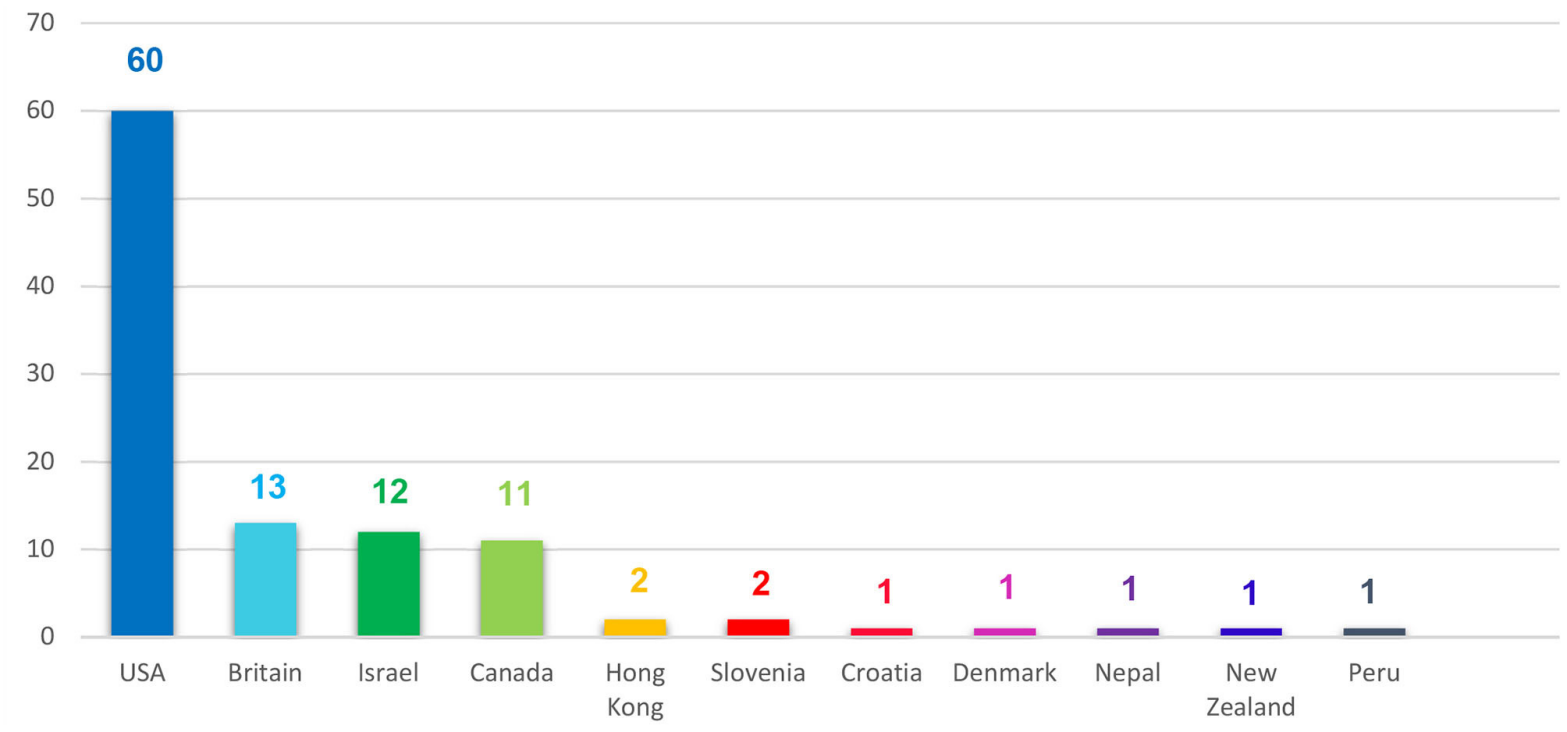
Geographic distribution of publications.

Regarding the geographical scope of the open studio approach to art therapy, the current research reveals that open studio models are mainly prevalent in North America and Europe (Figure 6). It is possible that our findings were influenced by the search languages (i.e., English and Hebrew); nevertheless, taking into account certain cultural and geo-political factors, they probably reflect the geographical prevalence of the open studio approach as well the global distribution of the art therapy profession and practice quite accurately.

### Participant Characteristics

**Figure 7:**
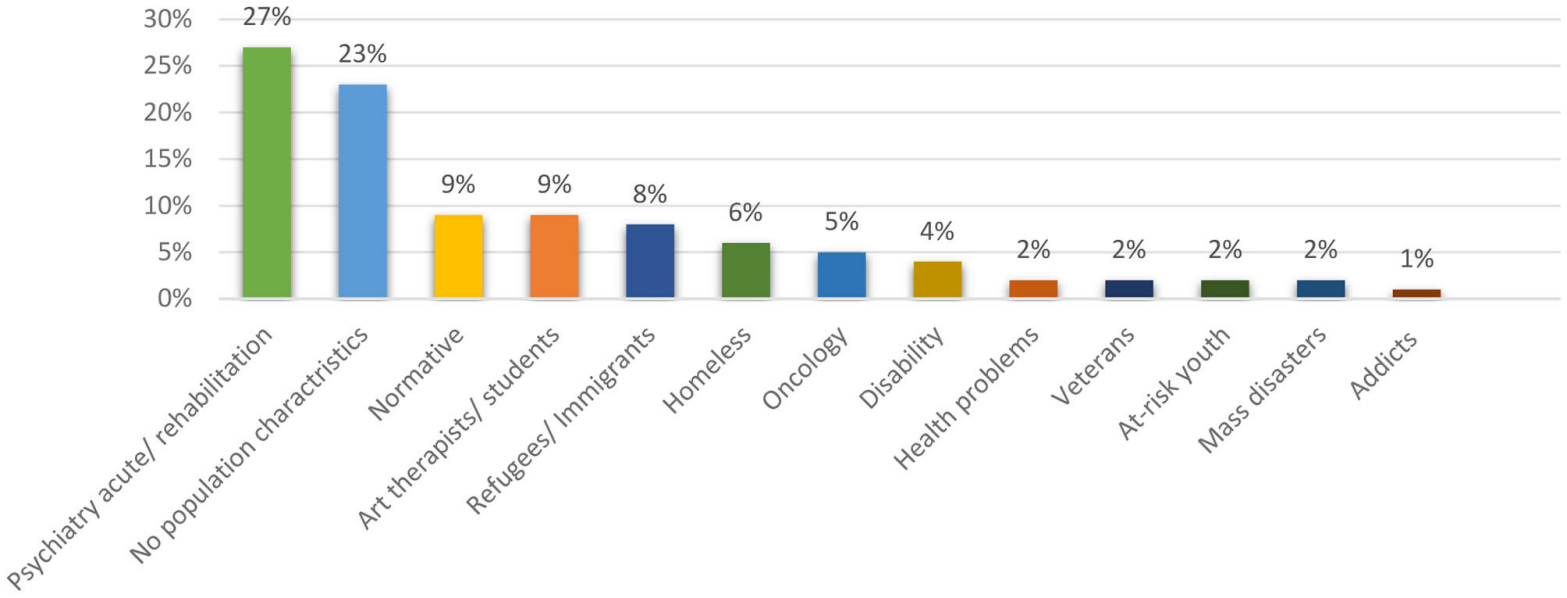
Distribution of results according to population characteristics.

Figure 7, which presents the populations among which the open studio approach to art therapy is practiced, reveals that 61% of the findings relate to populations that are coping with challenges; in a significant number of cases, the open studio model is implemented among populations dealing with psychiatric problems (27%). The open studio was also implemented among the following populations: immigrants and refugees (8%); the homeless (6%); oncology patients (5%); people with disabilities (4%) people with various health problems (2%) victims of mass disasters (2%); army veterans (2%) youth at-risk (2%) and drug-addicts (1%). 18% of the findings relate to normative populations, including a specific focus on students of art therapy and art therapists. 21% of the findings do not specify type of population.

**Figure 8:**
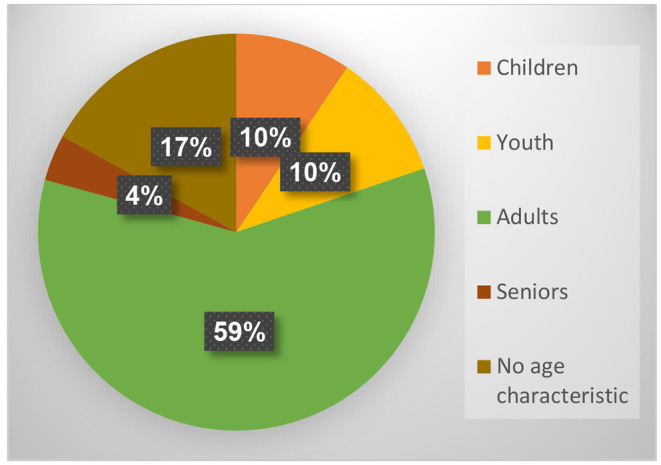
Distribution of results according to age.

In addition, we found that most of the texts relating to the open studio approach to art therapy focused on adults (59%). We also found that 10% of the publications relate to youth, 10% relate to children, and 4% to seniors (Figure 8). 17% of the findings do not specify ages or cannot be classified according to specific age criteria.

### Intervention Characteristics

**Figure 9:**
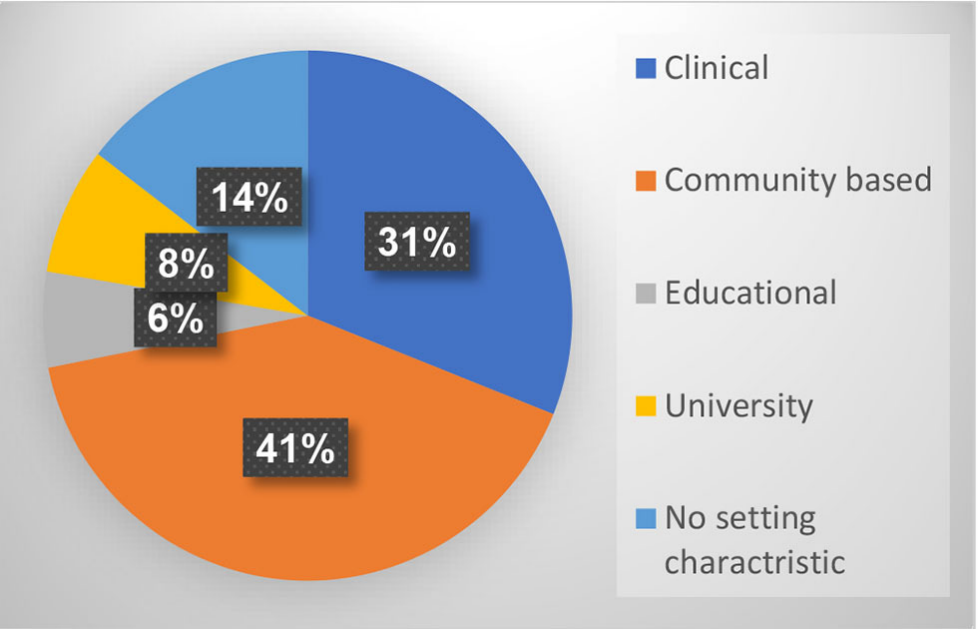
Distribution of results according to setting.

Figure 9, which presents the distribution of open studio settings, shows that most of the publications relate to community-based settings (41%), including art centers, churches, shelters for the homeless, rehabilitation centers for those suffering from mental health disorders, harm reduction centers, stores, galleries, museums, and more. 31% of the publications deal with an open studio in a medical setting, such as a psychiatric hospital, general hospital, clinic, and more. 8% focus on academic settings including use of the open studio model to train art therapists or to conduct academic research. 6% refer to educational settings, i.e., open studio programs in schools, and 14% of the publications do not refer to a specific setting.

Two main themes emerge from the findings: (a) the primary therapeutic role of art; and (b) the flexibility of the open studio model, enabling it to serve the needs of a wide range of populations in many different settings. Among this variety of uses, there are differences not only in population and setting, but also in regard to therapists' viewpoints. Table 2 presents these main differences between the various models in relation to: (**a) Group composition:** most of the findings that refer to the composition of the open studio group describe a changing group of participants. Groups of this kind can be found in psychiatric departments (Gonzalez-Dolginko, 2016), in cancer treatment centers (Piot and Plante, 2009) and in community-based models, where the studio is permanent and the participants come and go, and stay as much as they wish (drop in studio). Some open studio models serve a regular group of participants, such as in schools (Czamanski-Cohen, 2010) or training programs for art therapists, which are implemented for a specific period of time (Wix, 1995); (**b) The facilitator's role, which differs according to his or her therapeutic approach:** most findings that reference this aspect describe a model in which the facilitator does not engage in artmaking; rather, these facilitators concentrate on holding the space, witnessing or helping participants (Deco, 1998). The facilitators support the creative process (Braun, 1997) and allow for the birth of the artwork (Shapiro, 2014). One approach even suggests that the moderator should assume the role of the participant-artist's apprentice (Atkinson and D'Innocenzo, 2014); this idea stems from the belief that the artist knows best when it comes to his or her life and art. There are open studio approaches where the moderator works alongside participants as a partner (Marshall-Tierney, 2014) and models the creative process. In these cases, the status of moderator is equal to that of participant (Allen, 1995). These studios symbolize a shift from the hierarchy of therapist and patient to a more intersubjective approach based on equality (Allen, 1993). Another aspect of facilitation in the open studio is that often there is a group of facilitators holding the space. This strengthens the communality of this model, moreover it enables the facilitators to alternate different facilitation roles and participants to develop a relationship with the studio as a holistic entity that includes all that is in its space (Shapiro, 2014); (**c) Facilitators' perception of participants:** most of the findings that relate to the way open studio participants are perceived see participants primarily as “clients” or “patients” (e.g., Deco, 1998). This attitude prevails in clinical or community-based settings in which participants cope with mental health challenges. Other models of community- based settings stress aspects of individual welfare and social services and refer to participants as “participants.” The intention is that participants take part in the studio and engage in the creative process without being committed to an aesthetic outcome; the emphasis is on participation in the creative process and not the final product (e.g., Block et al., 2005). The perception of the participant as “artist” is characteristic of a minority of open studio models (e.g., Lentz, 2008) that emphasize aspects of health and normalization; this perception is more common among community-based models; (**d) Exhibiting artwork:** in most descriptions of the open studio, artwork is hung or exhibited in the studio space, but not outside the studio. In addition to the ethical need to maintain confidentiality, this convention stems from emphasis on the creative process that is characteristic of the therapeutic approach. Exhibition of the artwork may shift the focus to the quality and aesthetics of the outcome and undermine the authenticity and spontaneity of the creative process. In some models, exhibitions are organized for people outside the studio (Thompson, 2009), and some models encourage sale of the participants' artwork as part of the therapeutic process (Griffith et al., 2015). Exhibition and recognition of the artwork gives the artist a presence and voice within the social space and this is empowering and therapeutic in itself; (**e) Length:** the length of the studio session is geared to allow an in-depth process; most of the publications that refer to the length of the sessions describe sessions that range from 1.5 to 2 h (61%) or beyond that, 3–6 h (31%). Most models of open studios operate regularly in institutions or communities; however, some models are implemented for a limited time as a response to specific situations that demand therapeutic solutions, such as natural disasters (Linton, 2017). These circumstances sometimes dictate the need for a temporary, portable studio (Kalmanowitz and Lloyd, 2011). The internal structure that art therapists carry with them makes it possible for them to set up a portable studio in a wide variety of settings. This internal structure includes sensitivity and awareness of the power of artmaking and imagery, its ability to represent human experience, and the belief in human strength and resources. The patient is not perceived as helpless and the therapist is not seen as the one who has all the answers. Moreover, imagery is interpreted in more than one way to allow the therapeutic process to reach its full potential. Moreover, this vision enables an atmosphere that encourages expression, engagement in the creative process and a tapping of individual resources (Kalmanowitz and Lloyd, 2011); (**f) Theoretical foundation:** Ideological and political considerations stemming from professional perceptions of pathology and health determine whether a certain model classifies itself as art therapy, or not. Questions then arise as to what makes the open studio a model for art therapy and how does open studio practice position itself within the defined boundaries of the profession (e.g., Kapitan, 2008). A study of community studio art programs designed to empower people with disabilities and support their integration into society (Vick and Sexton-Radek, 2008) revealed that studios in the US define themselves differently than their European counterparts. European programs do not define themselves as art therapy; moreover, they emphasize the artistic talent of participants as well as the outcome. The US studios, in comparison, influenced by traditional art therapy principles, value process much more than the artistic talents of the participants reflected in the outcome. Facilitators in American settings tend to work on artistic projects alongside the participants, as partners and to enable modeling; in European programs, in comparison, moderators offer participants aesthetic and technical feedback on their work. Furthermore, studios for participants with disabilities are based on the assumption that people who are dealing with an ongoing situation in life are not sick; efforts made to destigmatize disability through activities that have social value and help reduce the experience of “otherness” (Vick and Sexton-Radek, 2008) reflect this attitude. The European programs tend to describe themselves as “not art therapy” due to what seems to be a narrow and outdated belief that the role of art therapy is to interpret the patient's artwork in order to find psychological or pathological meaning (Vick and Sexton-Radek, 2008).

**Table 2:** Distribution of open studio characteristics.

**Studio characteristic**	**Number of results relating to characteristic**	**Ways characteristic is manifestated and number of results for each manifestation**		
Therapeutic approach	59	Open studio is defined as art therapy - 45	Open studio is not defined as art therapy - 14	
Nature of the group	55	Changing group - 38	Permanent group - 17	
Therapist's role	53	Therapist does not engage in artmaking alongside the participants - 30	Therapist engages in artmaking alongside the participants - 23	
Perception of participants	61	Patient/client - 27	Participant - 24	Artist - 10
Art exhibition	56	Art work is not exhibited outside the studio - 32	Art work is exhibited outside the studio – 24	

Vick and Sexton-Radek (2008) assert that art therapy has moved away from a narrow medicine-based approach and taken of new professional, social and rehabilitative dimensions. This necessitates a redefinition of the therapeutic boundaries of art therapy.

## Discussion

Results show that most of the literature about the open studio is from the 1990's or later. This supports Wix's (2010) claim that studio practice has been overlooked in the documentation of art therapy history even though it has had a key role in the development of this field. Wix (2010) asserts that art therapy's bias toward its psychological rather than its artistic roots created lacunas in both theory and practice. She argues that bringing the studio history of art therapy to light will ground the profession in its artistic roots (Wix, 2010). The many and varied descriptions of the open studio approach in the literature enable identification of common core principles that are not dependent on setting or type of population. The open studio approach is grounded in the central role of art and an open and non-moderated creative process. The facilitator is responsible for holding the space in order to allow for individual expression in a group setting. The length of the session enables engagement in the creative process; it is also important that the studio is a liminal space (Timm-Bottos, 2016) and has a special atmosphere. These “art as therapy” approach-based principles define the appropriate physical place, therapeutic and artistic space, and session length that allow full engagement in the creative process.

Beyond its common core principles, the open studio is a flexible therapeutic model that can be implemented among different populations and in different settings. The literature shows there are different names for interventions that have the same principles, and this variety of names also reflects the shift in focus from one model to another, for example, open studio group (Deco, 1998), open studio process (Allen, 1995), community-based art studio (Vick and Sexton-Radek, 2008), therapeutic studio (Kapitan, 2008), art hive (Timm-Bottos, 2016), disability studio (Lentz, 2008), inhabited studio (Kalmanowitz, 2016), and portable studio (Kalmanowitz and Lloyd, 2011).

The following two examples serve to illustrate the diverse range of open studio components, which are presented in detail in the chapter on results. This variety of practices corroborates Shapiro's (2014) claim that the open studio is a dynamic entity and its unique character is shaped by the participants' specific needs, the features of each specific setting and the facilitators' worldview. The OSP (open studio process), for example, emphasizes the spiritual aspect of the studio by combining art and writing. In addition to the creative process, it utilizes two central elements, intention and witnessing, as fundamental mindfulness tools (Allen, 2016). Another example is the studio-based practice implemented by an artist collective that combines art and music in Kerrville State Hospital. It includes exhibition of the participants' work across a wide range of platforms, including art exhibitions, cultural events, quarterly art magazines, fashion shows, performance opportunities, and more. Through these displays of artistic creativity, individuals are recognized for their contribution to society, and this reduces stigma both inside and outside the walls of this psychiatric institution (Peterson and Etter, 2017).

From a theoretical perspective, the open studio incorporates Winnicot's (1958) concept of “the capacity to be alone in the presence of another,” which, in his view, is one of the most important indications of emotional maturity. Winnicott suggests that the capacity to be alone develops in the presence of a reliable mother. The constant and dependable studio environment, including physical organization of the space and the presence of the group and facilitators, creates trust and makes it possible for the participants to “be alone” and engage in an internal dialogue through the process of artmaking (Deco, 1998).

Allen (2016) explores spiritual aspects of the open studio, and describes an energy or life force that is manifested through the creative process and images that emerge when one engages in artwork alongside another person, an action which involves taking risk and being open to the unknown. Each participant has something unique to bring to life and to share with others (Allen, 2016). McNiff (1995) addresses the “transformative spirits” that are embodied in the images created in the studio. He stresses the importance of the group, as it is inherently more intelligent, creative and resourceful than any individual; moreover, it represents the empowering support of a community that is shaped by a multiplicity of participants and images. McNiff (1995) perceives the studio as a vessel of creative transformation in which the creative process heals and transforms life.

Wix (2010) relates to the studio as a location that enables integration: “These locations of fruitful interdependency invite the reason of the heart to integrate with the reason of the mind and to foster engagements with self, materials, and others” (p. 182). Shapiro (2014) suggests that transference occurs in the art studio setting; the object of this transference is the studio space itself, which can perhaps be seen as fulfilling a co-therapist role. Participants often attribute magical qualities to this therapeutic space because it enables the expression of their inner and imaginary worlds. Honig et al. (2019), who study the role of studio practice in an art therapy training program, propose expanding the therapist-client-art triad, which offers a foundation for understanding the potential space of art therapy, to a pentagonal model consisting of therapist, client, art, space and group. This expanded model can provide a deeper understanding of the potential space of the art studio and enable exploration of the relations between therapeutic dimensions. Space and group, in addition to the participant, art and facilitator, are central dimensions in the open studio approach and they expand our understanding of the contexts in which therapeutic and artistic processes take place.

Moreover, it seems that the studio approach to art therapy mirrors the expansion of art therapy to social and community spaces (Kapitan, 2008). Timm-Bottos (2016) asserts that art hives, which are based on the community art studio, purposely blur the distinctions between art therapy, art education, critical cultural studies, popular pedagogy, public science, and creative arts, and create a liminal place between home and institution that allows for healing, reparation and innovation. Crane and Byrne (2020) relate to the open studio as a third space in art therapy education, in which the students explore their own capabilities and limitations through practice with art materials, as they undergo a creative process in the presence of others. This enables them to discover inner wisdom and embodied knowledge that is grounded in practice. The open studio is offered as a liminal space between academic classes and the experience of clinical practice, in which students are free to take risks and make the connections in order to experience and witness transformation as it happens (Crane and Byrne, 2020).

## Limitations

This systematic scoping review has several limitations. First, because the search was done primarily in English and Hebrew, we may not have identified publications in other languages. However, as the term “open studio” is used in many disciplines, expanding the search terms to other languages would have created volumes of irrelevant data. In addition to English-language publications, the search identified nine publications in Hebrew, two publications in French and one in Spanish. If the search strategy had included the term “open studio” in other languages, additional publications may have come up in the search. In other words, we can assume that our findings do not represent all publications on the subject of the open studio approach to art therapy.

Another limitation relates to literature published before the digital era. It may be that articles published before the 1980's are not accessible through the systematic scoping of data bases, and earlier publications on the open studio approach may be missing from this present review. Nevertheless, it may also be that the studio approach to art therapy is less present in literature published before the 1980's due to trends in the development of the art therapy field (Malchiodi, 1995; Wix, 2010).

In addition, though this review includes chapters from books that are important to the thinking and practice of the studio approach, there is a possibility that not all book chapters were found and included in this review. Notably, publications of this kind are usually not included in systematic scoping reviews because they are difficult to locate.

Finally, as explained above, 14 relevant and yet unpublished findings were not included because we were unable to obtain them.

## Conclusions

This review reveals an increase in publications relating to the open studio over time, the prevalence of this approach, and the increasing implementation of its various models among diverse populations in many contexts. Several common principles guide the practice of this approach. They are (a) the central role of art and artmaking in the healing process; (b) the studio as an enabling space; (c) the importance of individual expression within the group or community space; and (d) the role of the facilitator as enabling and encouraging creative processes. Studies on the therapeutic efficacy of the open studio approach reveal that open studios make a positive contribution to the lives of participants (Table 1). As this is the first systematic scoping review of this field, we sought to map out existing literature and outline the main characteristics of the open studio approach. There is room for further expansion of this review to include additional languages.

Findings revealed that most of the research is based on qualitative or mixed method studies. A review of the limitations referenced in these studies reveals that generalizations are difficult to make because of small sample sizes and short time periods; no comparative studies were conducted between intervention and control groups. There is a need for additional research involving larger samples and a longer time frame, and for comparative studies of intervention and control groups. At a time when there is a preference for evidence-based therapeutic interventions, and despite the need for rigorous quantitative studies that can support assumptions that the open studio is an effective therapeutic intervention, there is a lack of meticulous, empirical research.

In-depth studies that examine open studio participants' processes and experiences, as well as the studio's components and how they contribute to the therapeutic process, can shed more light on how the open studio serves as a therapeutic intervention in art therapy. In addition, findings show that there are very few publications and studies on how the open studio serves children, youth and seniors. There is room for more research on the use of this approach among these populations. A final topic for research is the way in which the open studio influences the community in community-based settings, and whether encounters between members of the community within the open studio have an impact on the community outside the studio.

In summary, the strength of the open studio model is its inherent flexibility, which enables implementation in many different contexts among diverse populations. In addition, this model encourages individual development within a group or community. It may be that these features, the expansion of art therapy to social and community spaces and the central role of art in the therapeutic approach contribute to the increasing prevalence of this approach. Furthermore, the open studio approach may be an appropriate response to the new social challenges that have emerged in recent decades, such as refugee crises or natural disasters.

The open studio approach represents a return to the roots of the art therapy profession where art serves as a foundation for healing and psychological transformation, as well as a move beyond the walls of the psychiatric hospital, to social and community environments. As Kapitan claims, “The “studio” in the largest sense is perhaps an archetype or deep structure wanting to be made visible and re-connecting art therapists to the places and communities they wish to belong in some fundamental way” (Kapitan, 2003, p. 14). This return to the core principles of the art therapy profession counteracts the trend of “clinification” (Allen, 1992) that perhaps represented a desire to be affiliated with psychotherapy and the mental health professions. It may signify that the field of art therapy is undergoing a significant differentiation and individuation process. In addition, it may reveal the impact of recent community mental healthcare approaches, such as the salutogenic or healing and resilience models. These models resonate with the open studio approach that emphasizes the health, strength, creativity and empowerment of the participant-artist.

## Author Contributions

DF researcher and author. MB was the supervisor for this research and one of the reviewers in the process of the scoping review screening. All authors contributed to the article and approved the submitted version.

## Conflict of Interest

The authors declare that the research was conducted in the absence of any commercial or financial relationships that could be construed as a potential conflict of interest.

## References

[B1] Allan J. Barford H. Horwood F. Stevens J. Tanti G. (2015). ATIC: developing a recovery-based art therapy practice. Int. J. Art Ther. 20:14 10.1080/17454832.2014.968597

[B2] Allen P. B. (1983). Group art therapy in short-term hospital settings. Am. J. Art Ther. 22, 93–97. 10264528

[B3] Allen P. B. (1992). Artist in residence: an alternative to “clinification” for art therapists. Art Ther. 9, 22–29. 10.1080/07421656.1992.10758933

[B4] Allen P. B. (1993). Commentaries. Art Ther. 10, 192–193. 10.1080/07421656.1993.10759012

[B5] Allen P. B. (1995). Coyote comes in from the cold: the evolution of the open studio concept. Art Ther. 12, 161–166. 10.1080/07421656.1995.10759153

[B6] Allen P. B. (2016). Art making as spiritual path: the open studio process as a way to practice art therapy, in Approaches to Art Therapy: Theory and Technique (3rd ed.), ed Rubin J.A. (London: Routledge), 271–285.

[B7] Anthony W. A. (1993). Recovery from mental illness: The guiding vision of the mental health service system in the 1990s. Psychos. Rehab. J. 16, 11–23. 10.1037/h0095655

[B8] Atkinson E. D'Innocenzo L. M. (2014). Integration Art: A New Approach to Art Therapy. (Master's thesis). [Chicago (IL)]: The School of the Art Institute of Chicago.

[B9] Block D. Harris T. Laing S. (2005). Open studio process as a model of social action: a program for at-risk youth. Art Ther. 22, 32–38. 10.1080/07421656.2005.10129459

[B10] Braun L. N. (1997). In from the cold: art therapy with homeless men. Art Ther. 14, 118–122. 10.1080/07421656.1987.10759266

[B11] Chiu G. Hancock J. Waddell A. (2015). Expressive arts therapy group helps improve mood state in an acute care psychiatric setting. Can. Art Ther. Assoc. J. 28, 34–42. 10.1080/08322473.2015.1100577

[B12] Crane T. Byrne L. (2020). Risk, rupture and change: Exploring the liminal space of the Open Studio in art therapy education. Arts Psychother. 69:101666 10.1016/j.aip.2020.101666

[B13] Czamanski-Cohen J. (2010). “Oh! Now I remember”: the use of a studio approach to art therapy with internally displaced people. Arts Psychother. 37, 407–413. 10.1016/j.aip.2010.09.003

[B14] Czamanski-Cohen J. (2012). The use of art in the medical decision-making process of oncology patients. Art Ther. 29, 60–67. 10.1080/07421656.2012.680049

[B15] Deco S. (1998). Return to the open studio group: Art therapy groups in acute psychiatry, in: Art Psychotherapy Groups, eds Skaife S. Huet V. (London: Routledge), 100–107.

[B16] Fan C. Mak A. S. (1998). Measuring social self-efficacy in a culturally diverse student opulation. Soc. Behav. Pers. 26, 131–144.

[B17] Finn C. A. (2003). Helping students cope with loss: incorporating art into group counseling. J. Special. Group Work. 28, 155–165. 10.1080/714860157

[B18] Glinzak L. (2016). Effects of art therapy on distress levels of adults with cancer: a proxy pretest study. Art Ther. 33, 27–34. 10.1080/07421656.2016.1127687

[B19] Gonzalez-Dolginko B. (2016). Assigning meaning to art to optimize the patient experience in short-term psychiatry (L'attribution de sens à l'art pour optimiser l'expérience des patients en psychiatrie à court terme). Can. Art Ther. Assoc. J. 29:57 10.1080/08322473.2016.1233376

[B20] Griffith F. J. Seymour L. Goldberg M. (2015). Reframing art therapy to meet psychosocial and financial needs in homelessness. Arts Psychother. 46, 33–40. 10.1016/j.aip.2015.09.007

[B21] Heller A. (2015). The Contribution of “Open Studio” Program based Experiential -Learning to Developing Reflection Abilities and Self Efficacy of Elementary-School Children. (dissertation). [Cluj-Napoca (RO)]: Babes-Bolyai University.

[B22] Henley D. (1995). A consideration of the studio as therapeutic intervention. Art Ther. 12, 188–190. 10.1080/07421656.1995.10759158

[B23] Hogan S. (2001). Healing Arts: The History of Art Therapy. London: Jessica Kingsley.

[B24] Honig O. Rinat S. Feldman A. (2019). Studio Art Therapy as a Utopic Space (Structured or Open), a Space for Art Therapy Training and Analytic Group Art Therapy. Beit Berl College: Faculty of Arts – Hamidrasha.

[B25] Howells V. Zelnik T. (2009). Making art: a qualitative study of personal and group transformation in a community arts studio. Psychiatr. Rehab. J. 32, 215–222. 10.2975/32.3.2009.215.222 19136354

[B26] Kaimal G. Mensinger J. L. Drass J. M. Dieterich-Hartwell R. M. (2017). Art therapist-facilitated open studio versus coloring: differences in outcomes of affect, stress, creative agency, and self-efficacy (Studio ouvert animé par un art-thérapeute versus coloriage : différences de résultats sur l'affect, le stress, l'agentivi). Can. Art Ther. Assoc. J. 30, 56–68. 10.1080/08322473.2017.1375827

[B27] Kaimal G. Ray K. D. (2017). Free art-making in an art therapy open studio: changes in affect and self- efficacy. Arts Health. 9, 154–166. 10.1080/17533015.2016.1217248

[B28] Kalmanowitz D. (2016). Inhabited studio: Art therapy and mindfulness, resilience, adversity and refugees. Int. J. Art Ther. 21, 75–84. 10.1080/17454832.2016.1170053

[B29] Kalmanowitz D. Lloyd B. (2011). Inside- out Outside- in: Found Objects and Portable Studio, in Art in Action: Expressive Arts Therapy and Social Change, eds Levine E. G. Levine S. K. (London: Jessica Kingsley Publishers), 104–127.

[B30] Kapitan L. (2003). Re-enchanting Art Therapy: Transformational Practices for Restoring Creative Vitality. Springfield: Charles C Thomas.

[B31] Kapitan L. (2008). “Not Art Therapy”: revisiting the therapeutic studio in the narrative of the profession. Art Ther. 25, 2–3. 10.1080/07421656.2008.10129349

[B32] Klorer P. G. (2005). Expressive therapy with severely maltreated children: neuroscience contributions. Art Ther. 22, 213–220. 10.1080/07421656.2005.10129523

[B33] Kramer E. (2000). Art as Therapy. London: Jessica Kingsley Publishers.

[B34] Lentz R. (2008). What we talk about when we talk about art therapy: an outsider's guide to identity crisis. Art Ther. 25, 13–14. 10.1080/07421656.2008.10129355

[B35] Linton J. (2017). A natural response to a natural disaster: the art of crisis in nepal (Une réponse naturelle à une catastrophe naturelle : art de crise au Népal). Can. Art Ther. Assoc. J. 30:31 10.1080/08322473.2017.1317201

[B36] Malchiodi C. A. (1995). Studio approaches to art therapy. Art Ther. 12, 154–156. 10.1080/07421656.1995.10759151

[B37] Marshall-Tierney A. (2014). Making art with and without patients in acute settings. Int. J. Art Ther. 19, 96–106. 10.1080/17454832.2014.913256

[B38] Maselli R. L. (1998). An open studio pilot program: Art therapy in a health and wellness outreach ministry. (Master's thesis). [Pepper Pike (OH)]: Ursuline College.

[B39] Matsushima R. Shiomi K. (2003). Social self-efficacy and interpersonal stress in adolescence. Soc. Behav. Pers. Int. J. 31, 323–332.

[B40] McNiff S. (1995). Keeping the studio. Art Ther. 12, 179–183. 10.1080/07421656.1995.10759156

[B41] Moon C. H. (2016). Open studio approach to art therapy, in The Wiley Handbook of Art Therapy, eds. Gussak D. E. Rosal M. L. (Oxford; Malden: John Wiley & Sons, Ltd.), 112–121. 10.1002/9781118306543.ch11

[B42] Ourso L. K. (2016). Open Studio Art Therapy: A Participatory Study. (Master's thesis). [Saint Mary of the Woods (IN)]: Saint Mary-of-the-Woods College.

[B43] Peters M. Godfrey C. Khalil H. McInerney P. Parker D. Baldini Soares C. (2015). Guidance for conducting systematic scoping reviews. Int. J. Evid. Based Healthcare 13, 141–146. 10.1097/XEB.0000000000000050 26134548

[B44] Peterson J. Etter A. (2017). Creating community and shattering stigma: collaborative arts interventions for the forensic population (Création de communauté et réduction de la stigmatisation : interventions artistiques collaboratives avec des personnes bénéficiant de services de psych. Can. Art Ther. Assoc. J. 30:78 10.1080/08322473.2017.1381511

[B45] Phoenix-Beck H. (2018). Painting a Positive Mood: Open Studio Art With Older Adults. (Master's thesis). [Belmont (CA)]: Notre Dame de Namur University.

[B46] Piot V. Plante P. (2009). L'approach studio libre en oncologie: description de l'atelier d'art-therapie offert par maurice brault à la fondation québécoise du cancer (The open studio approach in oncology: A description of the art therapy workshop offered by Maurice Brault). Revue Québécoise Psychol. 30, 99–119.

[B47] Pitman A. (2016). Art as healing review. BJPsych Bull. 40:54 10.1192/pb.bp.114.049544

[B48] Prescott M. V. Sekendur B. Bailey B. Hoshino J. (2008). Art making as a component and facilitator of resiliency with homeless youth. Art Ther. J. Am. Art Ther. Assoc., 25, 156–163. 10.1080/07421656.2008.10129549

[B49] Sapouna L. Pamer E. (2014). The transformative potential of the arts in mental health recovery – an irish research project. Arts Health. 8, 1–12. 10.1080/17533015.2014.957329

[B50] Schwarzer R. Jerusalem M. (1995). Generalized self-efficacy scale, in Measures in Health Psychology: A User's Portfolio, Causal and Control Beliefs, eds Weinman J. Wright S. Johnston M. (Windsor: Nfer-Nelson), 35–37.

[B51] Seligman M. (2011). Flourish: A Visionary New Understanding of Happiness and Well-being. New York, NY: Free Press.

[B52] Shapiro J. (2014). Open studio: model for art therapy based on spontaneous creation process from an open, non-directing therapeutic approach, in Creation – The Heart of Therapy. ed Berger R. (Kiryat Bialik: Ach Publications), 135–158.

[B53] Sherir M. Maddux J. (1982). The self-efficacy scale: construction and validation. Psychol. Rep. 52, 663–671.

[B54] Stokrocki M. Andrews S. S. Saemundsdottir S. (2004). The role of art for homeless women and survivors of domestic violence. Visual Arts Res. 29, 73–82.

[B55] Thompson G. (2009). Artistic sensibility in the studio and gallery model: revisiting process and product. Art Ther. 26, 159–166. 10.1080/07421656.2009.10129609

[B56] Thompson G. (2016). Aesthetic Action and Self-Construction of an Artist Identity: The Impact of Art and Art Therapy on Subjectivity and Mental Illness in Qualitative Research. (dissertation). [New York (NY)]: Saybrook University.

[B57] Timm-Bottos J. (2016). Beyond counseling and psychotherapy, there is a field, i'll meet you there. Art Ther. 33, 160–162. 10.1080/07421656.2016.1199248

[B58] Timm-Bottos J. Reilly R. C. (2015). Neighborhood art hives: engaging communities in teaching and learning, in The SAGE Sourcebook of Service-Learning and Civic Engagement, eds. Delano-Oriaran O. Penick-Parks M. W. Fondrie S. (Thousand Oaks, CA: SAGE Publications) 179–184. 10.4135/9781483346625.n34

[B59] Tricco A. C. Lillie E. Zarin W. O'Brien K. K. Colquhoun H. Levac D.. (2018). PRISMA extension for scoping reviews (PRISMA-ScR): checklist and explanation. Ann. Intern. Med. 169, 467–473. 10.7326/M18-0850 30178033

[B60] Tyson E. H. Baffour T. (2004). Arts-based strengths: A solution-focused intervention with adolescents in an acute-care psychiatric setting. Arts Psychother. 31, 213–227. 10.1016/j.aip.2004.06.004

[B61] Vick R. M. Sexton-Radek K. (2008). community-based art studios in europe and the united states: a comparative study. Art Ther. 25, 4–10. 10.1080/07421656.2008.10129353

[B62] Watson D. Clark L. A. Tellegen A. (1988). Development and validation of brief measures of positive and negative affect: The PANAS scales. J. Pers. Soc. Psychol. 54, 1063–1070. 3397865 10.1037//0022-3514.54.6.1063

[B63] Winnicot D. W. (1958). The capacity to be alone, in Maturational Processes and the Facilitating Environment, eds Masud M. Khan R. (London: Hogarth).

[B64] Wix L. (1995). The intern studio: a pilot study. Art Ther. 12, 175–178. 10.1080/07421656.1995.10759155

[B65] Wix L. (2000). Looking for what's lost: the artistic roots of art therapy: mary huntoon. Art Ther. 17, 168–176. 10.1080/07421656.2000.10129699

[B66] Wix L. (2010). Studios as locations of possibility: remembering a history. Art Ther. 27, 178–183. 10.1080/07421656.2010.10129388

