# Supplementary material for: The Open Studio Approach to Art Therapy: A Systematic Scoping Review
Source: Front Psychol. 2020 Oct 20;11:568042. doi: 10.3389/fpsyg.2020.568042 (PMC7606997; doi:10.3389/fpsyg.2020.568042)
Supplement: Supplementary file 1 [file Table_1.DOCX]

Supplementary Material

# Results reference list

Adamson, E. (1984). Art as Healing. London: Coventure Ltd.

Alhajji, A., Dougherty, A., and Awais, Y.J. (2018). A Grant Proposal to Support a Mobile Art Studio for Arab Refugee Children [master’s thesis]. [Philadelphia (PA)]: Drexel University. Retrieved from https://www.worldcat.org/title/grant-proposal-to-support-a-mobile-art-studio-for-arab-refugee-children/oclc/1060575461&referer=brief_results

Allan, J., Barford, H., Horwood, F., Stevens, J., and Tanti, G. (2015). ATIC: Developing a recovery-based art therapy practice. International Journal of Art Therapy: Inscape. 20(1), 14. Retrieved from http://ezproxy.haifa.ac.il/login?url=https://search.ebscohost.com/login.aspx?direct=true&db=edb&AN=100776560&site=eds-live&scope=site

Allen, P. B. (1983). Group art therapy in short-term hospital settings. American Journal of Art Therapy. 22(3), 93-97.

Allen, P. B. (1992). Artist-in-Residence: An Alternative to “Clinification” for Art Therapists. Art Therapy. 9(1), 22–29. https://doi.org/10.1080/07421656.1992.10758933

Allen, P.B. (1993). Commentaries. Art Therapy. 10(4), 192–193. https://doi.org/10.1080/07421656.1993.10759012

Allen, P B. (1995a). Coyote comes in from the cold: The evolution of the open studio concept. Art Therapy. 12(3), 161–166. https://doi.org/10.1080/07421656.1995.10759153

Allen, P.B. (1995b). Art is a way of knowing: A guide to self-knowledge and spiritual fulfillment through creativity. Boston: Shambhala Publications.

Allen, P. B. (2008). Commentary on community-based art studios: underlying principles. Art Therapy. 25(1), 11–12. https://doi.org/10.1080/07421656.2008.10129350

Allen, P. B. (2014a). "Artist in Residence in the Studio of the Soul: A Quest for an Identity", in Identity and Art Therapy: Personal and Professional Perspectives, ed. M. B. Junge (Springfield, IL: Charles C Thomas Publisher), 59–67.

Allen, P. B. (2014b). "Intention and Witness: Tools for Mindfulness in Art and Writing", in Mindfulness and the Arts Therapies: Theory and Practice, ed. L. Rappaport (London; Philadelphia: Jessica Kingsley Publishers), 39–46.

Allen, P.B. (2016). "Art making as spiritual path: The open studio process as a way to practice art therapy", in Approaches to Art Therapy: Theory and Technique (3rd ed.), ed. J.A.Rubin (Routledge), 271–285. <https://doi.org/10.4324/9781315716015>

Baasch, K. (2013). "A self to call home: community-based art therapy and homelessness", in Using Art Therapy with Diverse Populations: Crossing Cultures and Abilities, eds. J. Kristel, S. Prasad, and P. Howie (London; Philadelphia: Jessica Kingsley Publishers), 201–207.

Block, D. and Gadiel, D. (1996). Commentaries. Art Therapy. 13(1), 4–5. https://doi.org/10.1080/07421656.1996.10759183

Block, D., Harris, T. and Laing, S. (2005). Open Studio Process as a Model of Social Action: A Program for At-Risk Youth. Art Therapy. 22(1), 32–38. Retrieved from http://ezproxy.haifa.ac.il/login?url=https://search.ebscohost.com/login.aspx?direct=true&db=eric&AN=EJ682611&site=ehost-live&scope=site

Braun, L. N. (1997). In from the cold: Art therapy with homeless men. Art Therapy. 14(2), 118–122. https://doi.org/10.1080/07421656.1987.10759266

Brooker, J., Cullum, M., Gilroy, A., Mccombe, B., Mahony, J., Ringrose, K. et al. (n.d.). The Use of Artwork in Art Psychotherapy with People Who are Prone to Psychotic States- An Evidence-Based Clinical Practice Guideline. Retrieved from www.goldsmiths.ac.uk

Buchalter, S. I. (2009). "Open Art Studio", in Art Therapy Techniques and Applications (London; Philadelphia: Jessica Kingsley Publishers), 153–160.

Cahn, E. (2000). Proposal for a studio-based art therapy education. Art Therapy. 17(3), 177–182. https://doi.org/10.1080/07421656.2000.10129696

Case, C., and Dalley, T. (1992). "The Studio- Based Open Group", in The Handbook of Art Therapy, (London; New York: Routledge), 196–199.

Chapin Stephenson, R. (2013). Promoting Well-Being and Gerotranscendence in an Art Therapy Program for Older Adults. Art Therapy. 30(4), 151–158. https://doi.org/10.1080/07421656.2014.846206

Chen Belo, I. (2015). Open Studio – A Therapeutic Intervention for Psychiatric Patients. 'Bar-Alon'- Abarbanel psychiatric hospital magazine. 9-10 (In Hebrew).

Chiu, G., Hancock, J. and Waddell, A. (2015). Expressive arts therapy group helps improve mood state in an acute care psychiatric setting. Canadian Art Therapy Association Journal. 28(1–2), 34–42. https://doi.org/10.1080/08322473.2015.1100577

Czamanski-Cohen, J. (2010). “Oh! Now I remember”: The use of a studio approach to art therapy with internally displaced people. The Arts in Psychotherapy. 37(5), 407–413. https://doi.org/10.1016/j.aip.2010.09.003

Czamanski-Cohen, J. (2012). The Use of Art in the Medical Decision-Making Process of Oncology Patients. Art Therapy. 29(2), 60–67. https://doi.org/10.1080/07421656.2012.680049

Deco, S. (1998). "Return to the open studio group: Art therapy groups in acute psychiatry", in: Art Psychotherapy Groups, eds. S. Skaife, and V. Huet (London: Routledge), 100-107.Retrieved from http://ezproxy.haifa.ac.il/login?url=https://search.ebscohost.com/login.aspx?direct=true&db=psyh&AN=1999-02517-004&site=ehost-live&scope=site

DeLucia, J. M. (2016). Art Therapy Services to Support Veterans’ Transition to Civilian Life: The Studio and the Gallery. Art Therapy. 33(1), 4–12. https://doi.org/10.1080/07421656.2016.1127113

Denegri Álvarez-Calderón, P. (2015). Terapia a través del arte usando el modelo de estudio abierto con un grupo de estudiantes de Psicología. [master’s thesis]. [Lima (PE)]: Pontifica Universidad Catolica Del Peru. Retrieved from http://tesis.pucp.edu.pe/repositorio/handle/20.500.12404/6529?show=full

Atkinson, E. & D’Innocenzo, L.M. (2014). Integration art: a new approach to art therapy. [master’s thesis]. [Chicago (IL)]: The School Of the Art Institute of Chicago. Retrieved from https://www.worldcat.org/title/integration-art-a-new-approach-to-art-therapy/oclc/881639381&referer=brief_results

Feldman, A. (2015). "The Open Studio – Development and Essence", in Open Studio in Art Therapy- The Israeli Art Therapy Graduate programs' Conference, Tel Aviv (In Hebrew).

Feen-Calligan, H. (2008). Service-learning and art therapy in a homeless shelter. The Arts in Psychotherapy. 35(1), 20–33. Retrieved from http://10.0.3.248/j.aip.2007.09.001

Franklin, M., Rothans, M. E. and Schpok, K. (2007). "Unity in Diversity Communal Pluralism in the Art Studio and the Classroom", in Art Therapy and Social Action, ed. F. Kaplan (London; Philadelphia: Jessica Kingsley Publishers), 213–230. Retrieved from https://search-ebscohost-com.ezproxy.haifa.ac.il/login.aspx?direct=true&db=nlebk&AN=180459&site=ehost-live&scope=site

Glinzak, L. (2016). Effects of art therapy on distress levels of adults with cancer: A proxy pretest study. Art Therapy. 33(1), 27–34. https://doi.org/10.1080/07421656.2016.1127687

Goebl-Parker, S. (2011). "Aesthetic Listening- A Reggio-Inspired Studio Research Paradigm for Art Therapy", in Art Therapy and Postmodernism: Creative Healing Through a Prism, ed. H. Burt (London; Philadelphia: Jessica Kingsley Publishers), 219–230.

Gonzalez-Dolginko, B. (2016). Assigning Meaning to Art to Optimize the Patient Experience in Short-Term Psychiatry (L’attribution de sens à l’art pour optimiser l’expérience des patients en psychiatrie à court terme). Canadian Art Therapy Association Journal. 29(2), 57. Retrieved from http://ezproxy.haifa.ac.il/login?url=https://search.ebscohost.com/login.aspx?direct=true&db=edb&AN=120040845&site=eds-live&scope=site

Griffith, F. J., Seymour, L., and Goldberg, M. (2015). Reframing art therapy to meet psychosocial and financial needs in homelessness. The Arts in Psychotherapy. 46, 33–40. https://doi.org/10.1016/j.aip.2015.09.007

Heller, A. (2015). The Contribution of "Open Studio" Program based Experiential -Learning to Developing Reflection Abilities and Self Efficacy of Elementary-School Children. [dissertation]. [Cluj-Napoca (RO)]: Babes-Bolyai University. Retrieved from https://www.worldcat.org/title/contribution-of-open-studio-program-based-experiential-learning-to-developing-reflection-abilities-and-self-efficacy-of-elementary-school-children/oclc/1043062851&referer=brief_results

Heller, A. (2018). "The Open Studio Program in Educational Settings", in When the Arts Therapies Meet the Educational System in Israel: Applicable Aspects, eds. S. Snir, and D. Regev (Haifa: Emili Sagol Creative Arts Therapies Research Center (CATRC), Graduate School of Creative Arts Therapies, Haifa University), 274–303. (In Hebrew).

Henley, D. (1995). A Consideration of the Studio as Therapeutic Intervention. Art Therapy. 12(3), 188–190. https://doi.org/10.1080/07421656.1995.10759158

Hogan, S. (2001) Healing arts: The history of art therapy. London: Jessica Kingsley. Retrieved from https://web-a-ebscohost-com.ezproxy.haifa.ac.il/ehost/ebookviewer/ebook/bmxlYmtfXzExNzU2Nl9fQU41?nobk=y&sid=b9410ec1-b350-4597-a163-4f6ef43cea8a@sessionmgr4008&vid=3&format=EB&rid=1

Honig, O., Rinat, S., and Feldman, A. (2019). Studio Art Therapy as a Utopic Space (Structured or Open), a Space for Art Therapy Training and Analytic Group Art Therapy. Beit Berl College: Faculty of Arts – Hamidrasha (In Press), (In Hebrew).

Howells, V., and Zelnik, T. (2009). Making art: A qualitative study of personal and group transformation in a community arts studio. Psychiatric Rehabilitation Journal. 32(3), 215–222. https://doi.org/10.2975/32.3.2009.215.222

Kaimal, G., Mensinger, J. L., Drass, J. M. and Dieterich-Hartwell, R. M. (2017). Art Therapist-Facilitated Open Studio Versus Coloring: Differences in Outcomes of Affect, Stress, Creative Agency, and Self-Efficacy (Studio ouvert animé par un art-thérapeute versus coloriage : différences de résultats sur l’affect, le stress, l’agentivi). Canadian Art Therapy Association Journal. 30(2), 56–68. https://doi.org/10.1080/08322473.2017.1375827

Kaimal, G., and Ray, K.D. (2017). Free Art-making in an Art Therapy Open Studio: Changes in Affect and Self- efficacy. Arts & Health. 9:2, 154-166. DOI: 10.1080/17533015.2016.1217248.

Kalmanowitz, D. (2016). Inhabited studio: Art therapy and mindfulness, resilience, adversity and refugees. International Journal of Art Therapy. 21(2), 75–84. https://doi.org/10.1080/17454832.2016.1170053

Kalmanowitz, D. & Ho, R. T. H. (2016). Out of our mind Art therapy and mindfulness with refugees, political violence and trauma. The Arts in Psychotherapy. 49, 57–65. https://doi.org/10.1016/j.aip.2016.05.012

Kalmanowitz, D. & Lloyd, B. (1999). Fragments of art at work: art therapy in the former Yugoslavia. The Arts in Psychotherapy. 26(1), 15–25. Retrieved from http://10.0.3.248/S0197-4556(98)00027-6

Kalmanowitz, D., and Lloyd, B. (2011). "Inside- out Outside- in: Found Objects and Portable Studio", in Art in Action: Expressive Arts Therapy and Social Change, eds. E. G. Levine and S. K. Levine (London; Philadelphia : Jessica Kingsley Publishers), 104–127. Retrieved from <https://search-ebscohost-com.ezproxy.haifa.ac.il/login.aspx?direct=true&db=nlebk&AN=387971&site=ehost-live&scope=site>

Kapitan, L. (2008). “Not Art Therapy”: Revisiting the Therapeutic Studio in the Narrative of the Profession. Art Therapy. 25(1), 2–3. https://doi.org/10.1080/07421656.2008.10129349

Kaplan, F. F.& Golub, D. (2005). Social action art therapy. Art Therapy. 22(1), 17–23. https://doi.org/10.1080/07421656.2005.10129467

Karkou, V. (2010). Arts Therapies in Schools: Research and Practice. London;Philadelphia: Jessica Kingsley Publishers.

Kimchi, S. & Lubin, I. (2017). The Open Studio Essence in Health Settings From the Art Therapist's perspective. Academic Journal of Creative Arts Therapies. 7(2), 696-706. (In Hebrew).

Langner, D. (2009). The Open Studio at Erie House: Using therapeutic art to increase self-esteem among at-risk youth. [dissertation]. [Chicago (IL)]: The Chicago School of Professional Psychology. Retrieved from http://ezproxy.haifa.ac.il/login?url=https://search.ebscohost.com/login.aspx?direct=true&db=psyh&AN=2009-99160-175&site=ehost-live&scope=site

Lentz, R. (2008). What we talk about when we talk about art therapy: An outsider’s guide to identity crisis. Art Therapy. 25(1), 13–14. https://doi.org/10.1080/07421656.2008.10129355

Levine, A. (1995). California art therapy trends: Evelyn Virshup, PhD (Ed.). The Arts in Psychotherapy. 22(1), 61–63. Retrieved from http://10.0.3.248/0197-4556(95)90023-3

Levy, B. A., Berberian, M., Brigmon, L. S. V., Gonzalez, S. N., and Koepfer, S. R. (2002). Mobilizing Community Strength: New York Art Therapists Respond. Art Therapy. 19(3), 106–114. https://doi.org/10.1080/07421656.2002.10129403

Linton, J. (2017). A Natural Response to a Natural Disaster: The Art of Crisis in Nepal (Une réponse naturelle à une catastrophe naturelle : art de crise au Népal). Canadian Art Therapy Association Journal. 30(1), 31. Retrieved from http://ezproxy.haifa.ac.il/login?url=https://search.ebscohost.com/login.aspx?direct=true&db=edb&AN=123641400&site=eds-live&scope=site

Luzzatto, P. (1997). Short-term art therapy on the acute psychiatric ward: the open session as a psychodynamic development of the studio-based approach. International Journal of Art Therapy. 2(1), 2–10. Retrieved from http://lib.haifa.ac.il/ill_pdf/ILL_OLD/10519.pdf

Luzzatto, P., and Gabriel, B. (2011). "Art Therapy", in Enhancing Cancer Care: Complementary Therapy and Support, ed. J. Barraclough (Oxford : Oxford University Press), 108-114. https://doi.org/10.1093/acprof:oso/9780199297559.003.0010

Lyddiat, E.M. (1971). Spontaneous Painting and Modelling: A Practical Approach in Therapy. London: Constable & Company Limited.

Malchiodi, C. A. (1995). Studio approaches to art therapy. Art Therapy. 12(3), 154–156. https://doi.org/10.1080/07421656.1995.10759151

Manchester, S. A. (2014). A qualitative study of art therapy open studios in the United States. [master’s thesis]. [Saint Mary of the Woods (IN)]: Saint Mary-of-the-Woods College.

Marshall-Tierney, A. (2014). Making art with and without patients in acute settings. International Journal of Art Therapy. 19(3), 96–106. https://doi.org/10.1080/17454832.2014.913256

Maselli, R. L. (1998). An open studio pilot program: Art therapy in a health and wellness outreach ministry. [master’s thesis]. [Pepper Pike (OH)]: Ursuline College. Retrieved from https://search.proquest.com/docview/304478572?accountid=14544

Matton, A., and Plante, P. (2014). Impact of displaying artwork in an open studio workshop offered to people in treatment for cancer. Canadian Art Therapy Association Journal. 27(1), 8–13. https://doi.org/10.1080/08322473.2014.11415591

McGraw, M. K. (1995). The art studio: A studio-based art therapy program. Art Therapy. 12(3), 167–174. https://doi.org/10.1080/07421656.1995.10759154

McNiff, S. (1995). Keeping the Studio. Art Therapy. 12(3), 179–183. https://doi.org/10.1080/07421656.1995.10759156

McNiff, S. (2014). "The Role of Witnessing and Immersion in the Moment of Arts Therapy Experience", in Mindfulness and the Arts Therapies: Theory and Practice, ed. L. Rappaport (London; Philadelphia: Jessica Kingsley Publishers), 30–38.

McNutt, J. V. (2013). "An open art studio model", in Art Therapy and Health Care, ed. C. A. Malchiodi (New York; London: Guilford Press), 281–290. Retrieved from http://ezproxy.haifa.ac.il/login?url=https://search.ebscohost.com/login.aspx?direct=true&db=psyh&AN=2012-32135-019&site=ehost-live&scope=site

Miller, G. G. (2016). Art Therapy in a Community Clinic Waiting Room: Examining Challenges and Effectiveness (Art-thérapie dans la salle d’attente d’une clinique communautaire : examen des défis et de l’efficacité). Canadian Art Therapy Association Journal. 29(1), 38. Retrieved from http://ezproxy.haifa.ac.il/login?url=https://search.ebscohost.com/login.aspx?direct=true&db=edb&AN=116102904&site=eds-live&scope=site

Moon, C. H. (2002). Studio art therapy: cultivating the artist identity in the art therapist. London; Philadelphia: Jessica Kingsley.

Moon, C. H. (2016). "Open Studio Approach to Art Therapy", in The Wiley Handbook of Art Therapy, eds. D.E. Gussak, and M. L. Rosal (Oxford; Malden: Jhon Wiley & Sons, Ltd.), 112–121.

Moon, C. H. & Shuman, V. (2013). "The community art studio: Creating a Space of Solidarity and Inclusion", in Using Art Therapy with Diverse Populations: Crossing Cultures and Abilities, eds. J. Kristel, S. Prasad, and P. Howie (London ;;Philadelphia: Jessica Kingsley Publishers), 194–200.

Morris, F. J. & Willis-Rauch, M. (2014). Join the Art Club: Exploring Social Empowerment in Art Therapy. Art Therapy. 31(1), 28–36. https://doi.org/10.1080/07421656.2014.873694

Nolan, E. (2019). Opening Art Therapy Thresholds: Mechanisms That Influence Change in the Community Art Therapy Studio. Art Therapy. 36:2, 77-85, DOI: 10.1080/07421656.2019.1618177

Orbach, N. (2019). Good Enough Studio: Material, Action and Space in Art Therapy and Education. Tel Aviv: Resling (In Hebrew).

Ourso, L. K. (2016). Open studio art therapy: A participatory study. [master’s thesis]. [Saint Mary of the Woods (IN)]: Saint Mary-of-the-Woods College.

Partridge, E. (2019). "Open studio setting", in Art Therapy With Older Adults: Connected and Empowered, ed. E. Partridge (London; Philadelphia: Jessica Kingsley Publishers), 47–56.

Pendleton, P. J. (1999). Painting A Path of Well-being: Art Therapy as a Link to Mental Health Treatment. Art Therapy. 16(1), 31–36. https://doi.org/10.1080/07421656.1999.10759348

Peterson, J., and Etter, A. (2017). Creating Community and Shattering Stigma: Collaborative Arts Interventions for the Forensic Population (Création de communauté et réduction de la stigmatisation : interventions artistiques collaboratives avec des personnes bénéficiant de services de psych. Canadian Art Therapy Association Journal. 30(2), 78. Retrieved from http://ezproxy.haifa.ac.il/login?url=https://search.ebscohost.com/login.aspx?direct=true&db=edb&AN=126670609&site=eds-live&scope=site

Phoenix-Beck, H. (2018). Painting a Positive Mood: Open Studio Art with Older Adults. [master’s thesis]. [Belmont (CA)]: Notre Dame de Namur University. Retrieved from <https://search.proquest.com/docview/1940274921?accountid=14544>

Piot, V., and Plante, P. (2009). L’approach studio libre en oncologie: Description de l’atelier d’art-therapie offert par Maurice Brault à la Fondation Québécoise du Cancer (The open studio approach in oncology: A description of the art therapy workshop offered by Maurice Brault). Revue Québécoise de Psychologie. 30(3), 99–119. Retrieved from <http://ezproxy.haifa.ac.il/login?url=https://search.ebscohost.com/login.aspx?direct=true&db=psyh&AN=2011-08356-007&site=ehost-live&scope=site>

Reyhani Dejkameh, M. and Shipps, R. (2018). From Please Touch to Art Access: The Expansion of a Museum-Based Art Therapy Program. Art Therapy. 35(4), 211–217. https://doi.org/10.1080/07421656.2018.1540821

Shapiro, J. (2014). "Open Studio: Model for Art Therapy Based on Spontaneous Creation Process from an Open, Non-Directing Therapeutic Approach", in Creation – The Heart of Therapy, ed. Berger, R. (Kiryat Bialik: Ach Publications), 135-158. (In Hebrew).

Shifron B., Giloah B., Shifron R., Asoulinand P., and Mizruhin A. (2012). The Contribution of a Therapeutic Studio to Preventing Recurrent Hospitalization of Patients with Schizophrenia. (In Press) (In Hebrew).

Stillerman-Cohen, S. (2013). The effects of participation in a community-based art studio on rehabilitation of participants with mental disorders. [master’s thesis]. [Beer Sheva (IL)]: Ben Gurion University of the Negev (In Hebrew).

Stokrocki, M., Andrews, S. S. and Saemundsdottir, S. (2004). The Role of Art for Homeless Women and Survivors of Domestic Violence. Visual Arts Research. 29(58), 73–82. Retrieved from http://ezproxy.haifa.ac.il/login?url=https://search.ebscohost.com/login.aspx?direct=true&db=psyh&AN=2004-14679-010&site=ehost-live&scope=site

Thompson, G. (2009). Artistic sensibility in the studio and gallery model: Revisiting process and product. Art Therapy. 26(4), 159–166. https://doi.org/10.1080/07421656.2009.10129609

Thompson, G. (2016). Aesthetic action and self-construction of an artist identity: The impact of art and art therapy on subjectivity and mental illness in qualitative research. [dissertation]. [New York (NY)]: Saybrook University. Retrieved from <http://ezproxy.haifa.ac.il/login?url=https://search.ebscohost.com/login.aspx?direct=true&db=psyh&AN=2016-99010-446&site=ehost-live&scope=site>

Timm-Bottos, J. (1995). ArtStreet: Joining Community through Art. Art Therapy. 12(3), 184–187. https://doi.org/10.1080/07421656.1995.10759157

Timm-Bottos, J. (2010). Reviews. Art Therapy. 27(3), 149–150. <https://doi.org/10.1080/07421656.2010.10129668>

Timm-Bottos, J., and Reilly, R. C. (2015). "Neighborhood art hives: engaging communities in teaching and learning", in The SAGE Sourcebook of Service-Learning and Civic Engagement, eds. O. Delano- Oriaran, M.W. Penick- Parks and S. Fondrie (Thousand Oaks, CA: SAGE Publications) 179–184. https://doi.org/10.4135/9781483346625.n34

Timm-Bottos, J. (2016). Beyond Counseling and Psychotherapy, There Is a Field. I’ll Meet You There. Art Therapy. 33(3), 160–162. https://doi.org/10.1080/07421656.2016.1199248

Timm-Bottos, J. (2017). Public Practice Art Therapy: Enabling Spaces Across North America (La pratique publique de l’art-thérapie : des espaces habilitants partout en Amérique du Nord). Canadian Art Therapy Association Journal. 30(2), 94. Retrieved from http://ezproxy.haifa.ac.il/login?url=https://search.ebscohost.com/login.aspx?direct=true&db=edb&AN=126670610&site=eds-live&scope=site

Vick, R. M. & Sexton-Radek, K. (2008). Community-Based Art Studios in Europe and the United States: A Comparative Study. Art Therapy. 25(1), 4–10. https://doi.org/10.1080/07421656.2008.10129353

Vick, R. M. (2016). "Community-based disability studios: Being and becoming", in The Wiley Handbook of Art Therapy, eds. D. E. Gussak and M. L. Rosal (Chichester, UK : Wiley), 829–839. Retrieved from http://ezproxy.haifa.ac.il/login?url=https://search.ebscohost.com/login.aspx?direct=true&db=psyh&AN=2016-25095-075&site=ehost-live&scope=site

Wadeson, H. (1996). When the Edges Bleed …. Art Therapy. 13(3), 208–210. https://doi.org/10.1080/07421656.1996.10759223

Wise, S. (2009). "Extending a hand: Open studio art therapy in a harm reduction center", in The Use of Creative Therapies With Chemical Dependency Issues, ed. S. L. Brooke (Springfield: Charles C Thomas), 37–50. Retrieved from http://ezproxy.haifa.ac.il/login?url=https://search.ebscohost.com/login.aspx?direct=true&db=psyh&AN=2009-18709-003&site=ehost-live&scope=site

Wix, L. (1995). The intern studio: A pilot study. Art Therapy. 12(3), 175–178. https://doi.org/10.1080/07421656.1995.10759155

Wix, L. (2000). Looking for What’s Lost: The Artistic Roots of Art Therapy: Mary Huntoon. Art Therapy. 17(3), 168–176. https://doi.org/10.1080/07421656.2000.10129699

Wix, L. (2010). Studios as locations of possibility: Remembering a history. Art Therapy. 27(4), 178–183. https://doi.org/10.1080/07421656.2010.10129388

Wu, M. F. (2006). The museum-based art education therapy project in New York’s Chinatown : the exploration of ethnic identity and adaptation among Chinese immigrant youth through art. [dissertation]. [Cincinnati (OH)]: Union Institute & University. Retrieved from https://www.worldcat.org/title/museum-based-art-education-therapy-project-in-new-yorks-chinatown-the-exploration-of-ethnic-identity-and-adaptation-among-chinese-immigrant-youth-through-art/oclc/959025455&referer=brief_results
